# Autologous hematopoietic stem cell transplantation significantly alters circulating ceramides in peripheral blood of relapsing-remitting multiple sclerosis patients

**DOI:** 10.1186/s12944-023-01863-7

**Published:** 2023-07-07

**Authors:** Aina Vaivade, Anna Wiberg, Payam Emami Khoonsari, Henrik Carlsson, Stephanie Herman, Asma Al-Grety, Eva Freyhult, Ulla Olsson-Strömberg, Joachim Burman, Kim Kultima

**Affiliations:** 1 Department of Medical Science, Clinical Chemistry, Uppsala University, Uppsala, Swede Sweden; 2 Department of Immunology, Genetics and Pathology, Uppsala University, Uppsala, Sweden; 3 Department of Biochemistry and Biophysics, Science for Life Laboratory, National Bioinformatics Infrastructure Sweden, Stockholm University, Solna, Sweden; 4 Department of Cell and Molecular Biology, Uppsala University, Uppsala, Sweden; 5 Division of Hematology, Uppsala University Hospital, Uppsala, Sweden; 6 Department of Medical Science, Neuroscience, Uppsala University, Uppsala, Sweden

**Keywords:** Lipidomics, Metabolomics, Autologous hematopoietic stem cell transplantation, Relapsing-remitting multiple sclerosis, Ceramides

## Abstract

**Background:**

The common inflammatory disease multiple sclerosis (MS) is a disease of the central nervous system. For more than 25 years autologous hematopoietic stem cell transplantation (AHSCT) has been used to treat MS. It has been shown to be highly effective in suppressing inflammatory activity in relapsing-remitting MS (RRMS) patients. This treatment is thought to lead to an immune system reset, inducing a new, more tolerant system; however, the precise mechanism behind the treatment effect in MS patients is unknown. In this study, the effect of AHSCT on the metabolome and lipidome in peripheral blood from RRMS patients was investigated.

**Methods:**

Peripheral blood samples were collected from 16 patients with RRMS at ten-time points over the five months course of AHSCT and 16 MS patients not treated with AHSCT. Metabolomics and lipidomics analysis were performed using liquid-chromatography high-resolution mass spectrometry. Mixed linear models, differential expression analysis, and cluster analysis were used to identify differentially expressed features and groups of features that could be of interest. Finally, in-house and in-silico libraries were used for feature identification, and enrichment analysis was performed.

**Results:**

Differential expression analysis found 657 features in the lipidomics dataset and 34 in the metabolomics dataset to be differentially expressed throughout AHSCT. The administration of cyclophosphamide during mobilization and conditioning was associated with decreased concentrations in glycerophosphoinositol species. Thymoglobuline administration was associated with an increase in ceramide and glycerophosphoethanolamine species. After the conditioning regimen, a decrease in glycerosphingoidlipids concentration was observed, and following hematopoietic stem cell reinfusion glycerophosphocholine concentrations decreased for a short period of time. Ceramide concentrations were strongly associated with leukocyte levels during the procedure. The ceramides Cer(d19:1/14:0) and Cer(d20:1/12:0) were found to be increased (*P* < .05) in concentration at the three-month follow-up compared to baseline. C16 ceramide, Cer(D18:2/16:0), and CerPE(d16:2(4E,6E)/22:0) were found to be significantly increased in concentration after AHSCT compared to prior to treatment as well as compared to newly diagnosed RRMS patients.

**Conclusion:**

AHSCT had a larger impact on the lipids in peripheral blood compared to metabolites. The variation in lipid concentration reflects the transient changes in the peripheral blood milieu during the treatment, rather than the changes in the immune system that are assumed to be the cause of clinical improvement within RRMS patients treated with AHSCT. Ceramide concentrations were affected by AHSCT and associated with leukocyte counts and were altered three months after treatment, suggesting a long-lasting effect.

**Supplementary Information:**

The online version contains supplementary material available at 10.1186/s12944-023-01863-7.

## Introduction

The autoimmune disorder multiple sclerosis (MS) is known to affect the central nervous system (CNS). The two main processes driving this disease are immune-mediated inflammatory demyelination and neurodegeneration with axonal loss [1]. The most common form of MS is relapsing-remitting multiple sclerosis (RRMS) which is characterized by relapses followed by complete or near-to-complete recovery. Over time the recoveries may become incomplete resulting in the accumulation of disability. Around 20% of RRMS subjects later develop secondary-progressive multiple sclerosis (SPMS), where the disease progression is dominant, and the relapses occur on the background of it. Finally, 5 to 15% of MS patients have primary progressive MS, characterized by the gradual progression of disability from disease onset [2, 3].

Lipids are a diverse group of molecules that have important roles in various different metabolomic pathways [4]. Alternations in lipid metabolomic pathways, such as the sphingolipid (SP) metabolism, have previously been suggested to play a crucial role in the pathomechanism of MS [1, 5]. Ceramides, a major subclass of SPs, have been found to alter in MS lesions, cerebrospinal fluids, and plasma of MS patients. In addition, several ceramides are known to be altered in serum of MS patients compared to healthy controls, and it has been suggested that these altered levels may be associated with physical disability and retinal neurodegeneration [5]. Due to the sensitivity and specificity of mass spectrometry, it is a good method of choice when analyzing the assembly of lipids within an individual, known as the lipidome [6]. By using a nontargeted approach the major parts of the lipidome can be quantified, and thus unknown but relevant lipids are quantified, making it the most powerful tool for identifying novel lipid biomarkers or mediators [7].

Autologous hematopoietic stem cell transplantation (AHSCT) is a widely established procedure mainly used to treat blood malignancies. The use of AHSCT in treating autoimmune diseases, including MS, has increased in recent years [8–10]. It has been shown that treatment with AHSCT is highly effective in suppressing the inflammatory activity in MS. The treatment can lead to neurological improvement in patients with RRMS [11], resulting in the alleviation of symptoms, improved disability, and maintained remission. However, for patients with progressive MS AHSCT has a temporary effect, and the disease symptoms return over time [12]. It is thought that AHSCT leads to an immune system reset where the new system is more tolerant; however, the exact mechanism is not well characterized [13]. Factors influencing the success of this treatment are disease duration, the severity of disability, and patients’ age [12].

Proteomics analysis on RRMS patients undergoing AHSCT have previously shown that the treatment results in large, but short-lasting, changes in serum levels of proteins involved in inflammatory procedures. However, persistent impact of AHSCT on the inflammatory environment in peripheral blood was not found [14]. To further understand how AHSCT affects biological processes, the effects on circulating metabolites and lipids in peripheral blood from RRMS patients undergoing AHSCT was investigated, which may be important for the long-lasting effects seen with the treatment.

## Materials and methods

### Patients

This study included all sixteen RRMS patients treated with AHSCT from 2015 to 2016 at Uppsala University Hospital, Sweden. All patients had high inflammatory activity at disease onset, first-line drugs were insufficient, and other treatments were considered inappropriate. Further, all participants had an active disease state regardless of treatment attempts with at least one disease-modifying drug. In addition, 16 controls consisting of newly diagnosed RRMS patients are included in this study.

Patients undergoing AHSCT were clinically followed-up for three years after transplantation. The clinical data includes the Expanded Disability Status Scale (EDSS), occurring relapses, MRI activity, and no evidence of disease activity (NEDA-3) regarding the three parameters; clinical relapses, confirmed disease worsening, and MRI events (Supplementary, Table S1).

### Sample collection

**Fig. 1:**
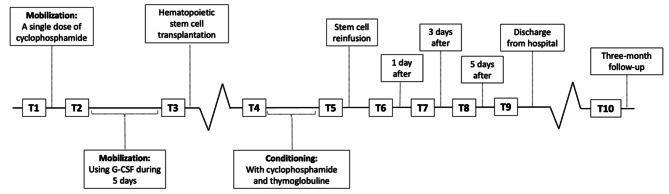
Timeline for AHSCT, including the ten-time points for blood sampling. (T1) prior to mobilization, (T2) following mobilization using cyclophosphamide, (T3) stem cell collection, (T4) prior to conditioning, (T5) prior to SCR, (T6) + 1 day after SCR, (T7) + 3 days after SCR, (T8) + 5 days after SCR, (T9) hospital discharge and (T10) the three-month follow-up

Peripheral blood samples were collected from 16 RRMS patients treated with AHSCT at Uppsala University Hospital, Sweden, from 2015 to 2016. The procedure used for stem cell collection and transplantation has been described previously by Wiberg et al. 2020. In brief, the peripheral blood samples were collected at ten timepoints (Fig. 1): (T1) prior to mobilization, (T2) following mobilization with cyclophosphamide, (T3) timepoint of stem cell collection, (T4) prior to the conditioning regime with cyclophosphamide and thymogobuline, (T5) prior to stem cell reinfusion (SCR), (T6) + 1 day after SCR, (T7) + 3 days after SCR, (T8) + 5 days after SCR, (T9) at the time of hospital discharge and (T10) at the three-month follow-up. After centrifuging the whole blood samples at 2400 g for seven minutes and allowing them to coagulate, the serum was stored at -80℃ in sterile polypropylene tubes.

### Chemicals

Methanol (≥ 99.9%, high-resolution mass spectrometry (HRMS) grade) was obtained from Honeywell (Seelze, Germany). From VWR Chemicals (Leuven, Belgium) acetonitrile (≥ 99.9%, HRMS grade) and isopropanol (≥ 99.9%, HRMS grade) were obtained. Formic acid (American Chemical Society reagent grade) and acetic acid (100%, anhydrous for analysis) was obtained from Merck (Darmstadt, Germany). The Milli-Q IQ 7000 system (Merck Millipore, Burlington, MA, USA) was used for water purification. Deuterium-labeled internal standards were supplemented to all samples as described previously [15–17] and specified in the Supplementary (Table S2 and S3).

### Sample preparation

All samples were prepared separately for metabolomic and lipidomic analysis using different precipitation solutions. For metabolite extraction (metabolomics), a precipitation solution made from methanol supplemented with 45 dissolved internal standards was used (Table S2). For lipid extraction (lipidomics), a precipitation solution made from isopropanol supplemented with 18 dissolved internal standards was used (Table S3).

Serum samples were thawed on ice, after which 50 µL serum was combined with 150 µL ice-cold precipitation solution in a 1.5 mL Eppendorf tube. After vortexing the mixture for 15 s it was stored at -20 °C for 60 min. After being centrifuged at 21 100 g and 4 °C, 100 µL of the supernatant were transferred into a glass vial suitable for high-performance liquid chromatography (HPLC) and stored at -80 °C until the time of analysis.

Each batch contained up to 23 samples, and in total eight batches were prepared. Finally, 10 µL aliquots from each sample were pooled into a batch pool. The grand pool, created by pooling all batch pools, contained all samples and was used throughout the experiment as a quality control (QC).

### Liquid chromatography - high-resolution mass spectrometry analysis

The metabolomics and lipidomics experiments were performed using different liquid chromatography HRMS (LC-HRMS) methods but with the same experimental design. Prior to sample injection, 20 to 30 injections of QC were used to precondition the column and were excluded in data evaluation. The patient samples were injected in a randomized order while maintaining the grouping of samples from each patient. Depending on the number of samples per patient a QC followed by a blank was injected every 9th to 14th sample.

The LC-HRMS analyses have previously been described in detail [15, 17]. In brief, two µL of each sample was injected on reversed-phase HPLC columns using an Ultimate 3000 HPLC system from Thermo Scientific that was connected to a high-resolution hybrid quadrupole Q Exactive Orbitrap MS the same manufacturer. The column used for the metabolomics analysis was Accucore aQ C18 100 × 2.1 mm, 2.6 μm, while Accucore C30 100 × 2.1 mm, 2.6 μm was used for the lipidomics analysis, both from Thermo Scientific. The chromatographic gradient with increasing percentage of organic solvents used for metabolomics was mobile phases H_2_O with 0.1% formic acid (mobile phase A) and 90% acetonitrile, 10% isopropanol with 0.1% formic acid (mobile phase B). For lipidomics the gradient was 60% H_2_O, 40% methanol with 0.1% acetic acid (mobile phase A), and 90% isopropanol, 10% methanol, and 0.1% acetic acid (mobile phase B). The analyses of all samples were performed in full scan mode at resolution 70,000 in the positive ionization mode (for metabolomics and lipidomics) and negative ionization mode (only used for lipidomics with separate injections for the negative mode) scanning the m/z ranges m/z 70–900 for metabolomics and m/z 100–1200 for lipidomics. Subsequent MS/MS analyses were performed on the eight batch pools of the samples using data-dependent acquisition at resolution 35,000 with the same chromatographic settings and tune parameters as the full scan experiments. The following normalized collision energies (NCE) were used, with separate injections at each NCE: for metabolomics NCE = 20, 30, and 40, for lipidomics NCE = 10, 20, 30, 40, and 50.

### Statistical methods

Raw data was centroided and converted to mzML using msConvert [18]. The peak-picked data was preprocessed using the KNIME platform [19] following this pipeline: FeatureFinderMetabo [20] was used for data quantification, and feature linking across samples was performed with featureLinkerUnlabelledQT, using non-default parameters (Tables S4 and S5).

The software environment R v3.6.1 [21] was used for statistical analysis. To stabilize the variance, the intensities were transformed to a log_2_ scale. Features with less than 70% coverage in QC and patient (undergoing AHSCT) samples were excluded from further analysis. Patient and QC data was normalized using loess-regression normalization (span value of 0.2) with the *loessFit* function from the *limma* package [22]. Followed by blank filtering by which features with an average signal of > 1% in blank injections were removed. In the final filtering step, all features with a coefficient of variation (CV) above 30% in QCs were excluded from further analysis.

For data quality control, a multilevel principal component analysis (PCA) was performed (scaled and centered) on timepoints T1 to T10 using the *pca* function from the *mixOmics* package [23] with patient id as multilevels.

### Differential expression analysis

To extract differentially expressed features through AHSCT (T1 to T10), repeated-measures analysis of variance (ANOVA) (Anova function from the car package [24]) was performed using a linear mixed-effect model (*lme* function from R-package *nlme* [25]). Patient id was set as a random effect and features as response variables. Timepoint, age, sex, and BMI were considered covariates. For the 16 newly diagnosed patients BMI was imputed. *P* values related to timepoints were false discovery rate (FDR) adjusted, and an FDR value below 0.001 was considered statistically significant.

For visualization purposes, the extracted differentially expressed features were subjected to a multilevel PCA (scaled and centered) using the *pca* function [23] with patient id as multilevels. Linear mixed-effect models and ANOVA were used to evaluate which PC (PC1 to PC10) described the differential expression the most and hence used for the plot. PCs with a *P* value below 0.05 were considered statistically significant.

To evaluate how feature levels changed after AHSCT (T1 to T10), adjacent timepoints were compared, as well as the three-month follow-up (T10) versus baseline (T1). Further T1 and T10 were compared with newly diagnosed RRMS. To calculate the fold changes and their *P* values the computed estimated marginal means (EMMs) were used together with the *contrast* function. The EMMs were calculated using the *emmeans* function from R-package *emmeans* [26] and were based on the linear mixed-effect model. For visualization, hierarchical clustering was performed on the foldchanges using *pheatmap* [27] with ward.D2 as clustering method and the similarity measure Euclidean distance. Clusters with an Approximately Unbiased (AU) *P* value ≥ 0.95 were considered significant. To investigate if any lipid could indicate the outcome of AHSCT, lipid levels prior to treatment were compared between patients with NEDA-3 after three years and patients with disease activity within this time span.

### Lipid and metabolite association to clinical measurements

To investigate the association between the measured metabolome and lipidome to basic clinical data, the association between features and the following six clinical measurements were investigated; body temperature, C-reactive protein (CRP), erythrocytes, leukocytes, thrombocytes, and neutrophils. To extract associated features of repeated measurements linear mixed-effect model was created as described for the differential expression analysis, with respective clinical measurements as covariates. *P* values were FDR adjusted and features with an FDR below 1e-7 were used for further analysis.

To visualize the associations -log_10_-transformed *P* values were multiplied by the coefficient’s direction. Using *pheatmap* hierarchical clustering was performed with ward.D2 as the clustering method and Euclidean distance as similarity measurement.

### Feature identification and post-mass calibration

For the identification of lipidomics features several approaches were used; an in-house compound library, the LIPID MAPS database [28], and in silico library. These in silico libraries were created using the nf-core workflow metaboigniter [29]. The parameters used for CSIFingerID [30–32] can be found in Supplementary Table S5. When matching features to the libraries +/- 8 ppm and +/- 10 s in retention time was used based on internal standards. In addition, the generated CANAPOUS [33, 34] file from SIRIUS was used for feature classification. Metabolomic features were identified by matching (+/- 10 ppm and +/- 10 s in retention time) with a previously created library. The targeted MS/MS data was identified using CSIFIngerID (Supplementary Table S5). A post-mass calibration on the identified features was performed to evaluate the quality of the matches in the lipidomics data, resulting in a standard deviation (SD) of 1.08 ppm mass error in positive mode and 0.98 in negative mode (Supplementary Figure S1).

### Enrichment analysis

Enrichment analysis on the lipidomics data was done using MetaboAnalyst [35]. This analysis was performed on all groups of interest from the differential expression analysis and for the lipids associated with one of the six clinical measurements. Due to too few IDs, it was not possible to perform enrichment analysis on the metabolomics data.

## Results

### Effects of AHSCT on the lipidome and metabolome of RRMS patients

**Fig. 2:**
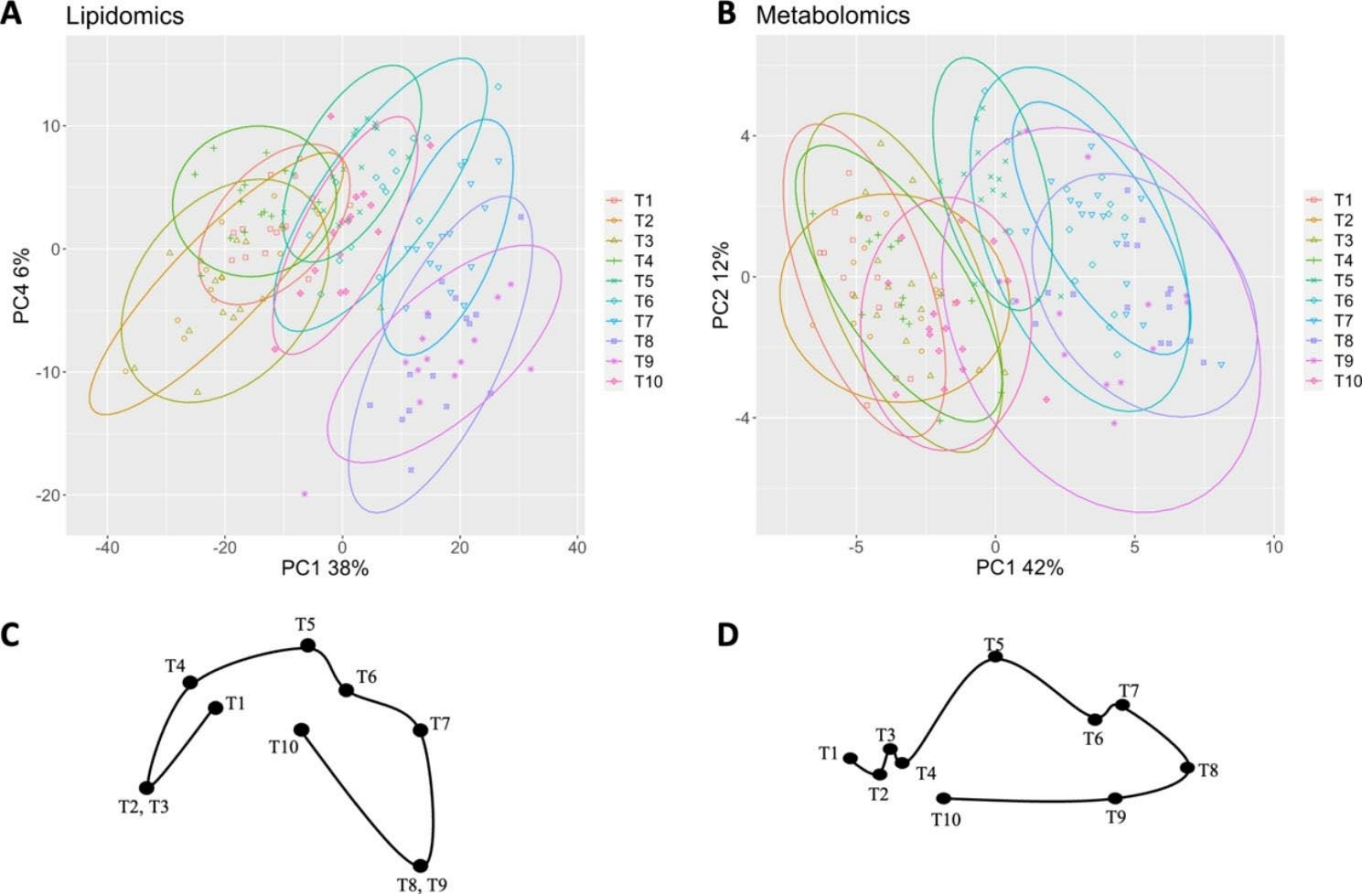
PCA depicting PC1 and PC4 obtained from differentially expressed features, (A and C) lipidomics, and (B and D) metabolomics, from RRMS patients undergoing AHSCT. The percentage of explained variance by each PC is presented on the corresponding axis. The timepoints (T1 to T10) for blood sampling are described in Fig. 1

We first investigated which features were differentially expressed (q < 0.001) through the course of or after AHSCT (T1 to T10) resulting in 657 features in the lipidomics and 34 in the metabolomics dataset. A multilevel PCA was performed on the features. To visualize the variation of these feature concentrations through AHSCT, linear mixed-effect models were fitted to the PCA scores and PC1 and PC4 for lipidomics, and PC1 and PC2 for metabolomics dataset were significant (*P* < .05) (Fig. 2). Administration of cyclophosphamide (T2) and granulocyte colony stimulating factor (G-CSF) (T3) during mobilization was associated with minor changes in lipid and metabolite concentrations. During the approximately three to five weeks prior to initiation of the conditioning regime (T4) the analyte concentrations almost returned to their initial concentrations (T1). The main alterations in lipid and metabolite concentrations were observed immediately after conditioning with cyclophosphamide and thymoglobuline (T5) and continued until hospital discharge (T9). Three months after treatment (T10), lipid and metabolite concentrations were almost back to initial concentrations (T1).

**Fig. 3:**
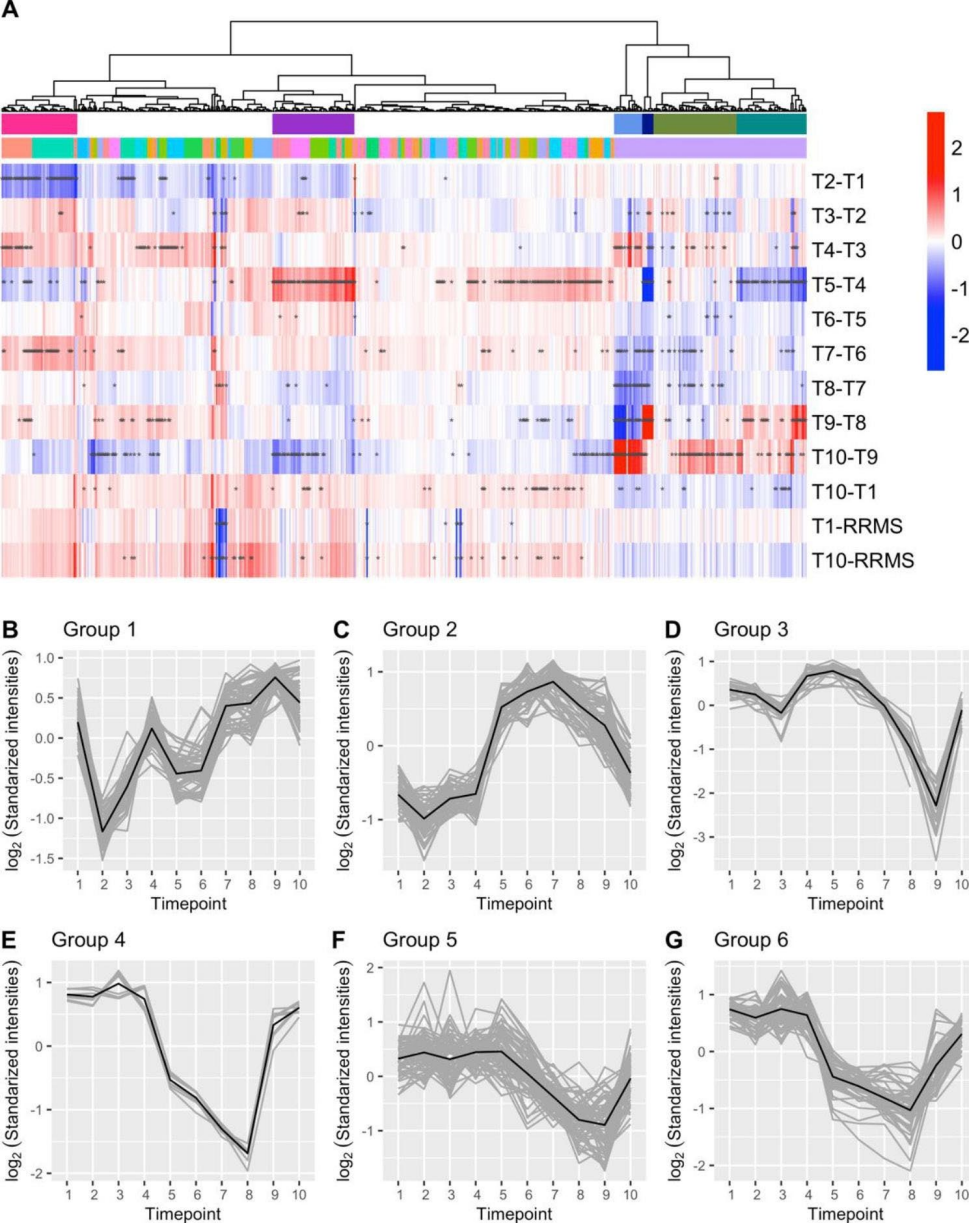
Expression profiles of differentially expressed features from the lipidomics dataset. (A) Heatmap of the log_2_(fold change) for adjacent sampling timepoints during AHSCT for RRMS patients’ lipidomics data, including the log_2_(fold change) for the three-months follow-up (T10) and baseline (T1) comparison. In addition, T1 and T10 are compared to newly diagnosed RRMS patients (RRMS in the figure). The red gradient indicates a positive log_2_(fold change), and the blue indicates a negative log_2_(fold change). Significant (P < .05) log_2_(fold change) is marked with an asterisk. (B to G) The expression profiles of the six groups of interest. The gray lines represent the mean expression profile for each feature, while the black line represents the mean expression profile for all features within one group

The hierarchical clustering of differentially expressed features resulted in 80 significant clusters (AU *P* value ≥ 0.95) for the lipidomics data and five for the metabolomics data (Supplementary, Figure S2). For further analysis of the lipidomics dataset six clusters of interest were extracted (Fig. 3), referred to as groups one to six (G1 - G6). The detailed lipidomics enrichment analysis results, including boxplots of identified lipids, are presented in Supplements Table S7 to S11 and Figures S3 to S7.

### Cyclophosphamide administration during mobilization was associated with a significant decrease in glycerophosphoinositol concentrations

**Fig. 4:**
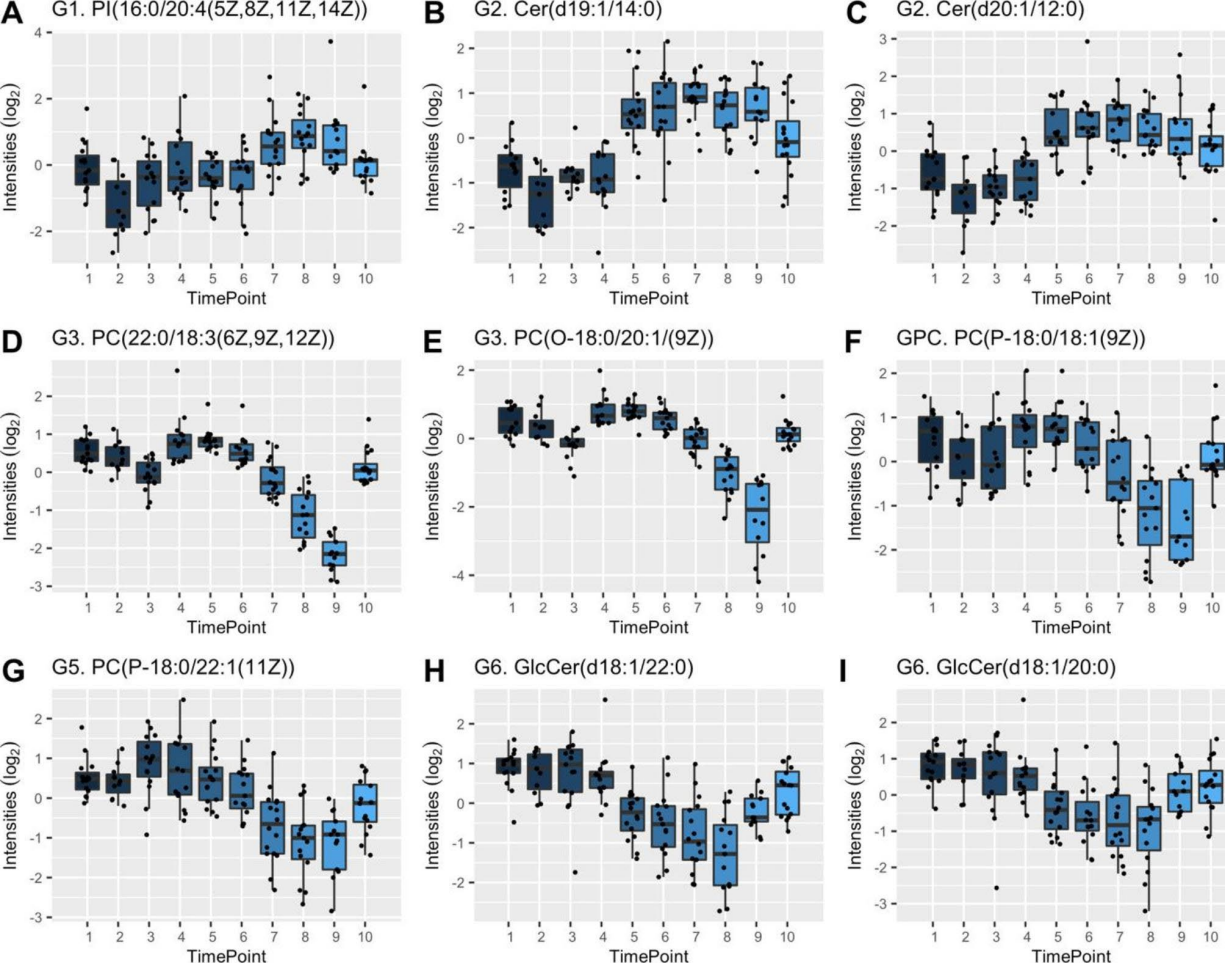
Boxplots of identified lipids in groups Group 1 to 6 (G1-G6). Lipids were chosen as representatives of the different classes

Glycerophosphoinositols (GPI) (including the lipid PI(16:0/20:4(5Z,8Z,11Z,14Z)), Fig. 4A) were found to be significantly enriched (*P* < .01) in Group 1 (Fig. 3; Table 1). During the first step of the mobilization, a single dose of cyclophosphamide was administered (T2) which was associated with a significant (*P* < .05) decrease in GPI concentration. Interestingly, when the conditioning regime (T5) where cyclophosphamide and thymoglobuline are administered, no effect was observed. Analyte concentrations were increased at T7, three days after the hematopoietic SCR. Finally, at the three-month follow-up (T10), GPI concentrations were restored with no significant differences compared to before treatment initiation.

### The conditioning regime was associated with an increase in ceramide and glycerophosphoethanolamine concentrations

The two lipid classes, ceramides, and glycerophosphoethanolamine (GPE), were found to be significantly enriched (*P* < .01) in Group 2 (Fig. 3; Table 1). The administration of cyclophosphamide during mobilization (T2) was typically associated with a minor decrease in lipid concentrations. This decrease was found to be significant (*P* < .05) for the ceramide Cer(d20:1/12:0) (Fig. 4C). Surprisingly, thymoglobuline and cyclophosphamide administration during conditioning was associated with significant and substantially increased lipid concentrations (T5) for both lipid classes. For the ceramides Cer(d19:1/14:0) and Cer(d20:1/12:0), the concentrations were significantly (*P* < .05) increased at the three-month follow-up, thus demonstrating lasting effects (Fig. 4B to C).

### Hematopoietic SCR was associated with decreased glycerophosphocholine concentrations

The lipid class glycerophosphocholine (GPC) was found to be significantly enriched (*P* < .01) in two groups; Groups 3 and 5 (Fig. 3; Table 1). Four of the identified lipids were: PC(22:0/18:3(6Z,9Z,12Z)) and PC(O-18:0/20:1(9Z)) for Group 3, and PC(16:0/O-16:0) and PC(P-18:0/22:1(11Z)) for Group 5 (Fig. 4D-G). For GPC within Group 3 the mobilization with cyclophosphamide (T2) and G-CSF (T3) was associated with a more pronounced decrease in lipid concentration compared to Group 5. The following hematopoietic stem cell collection (T4) was associated with increased lipid concentrations. For both Group 3 and 5 hematopoietic SCR (T6) was associated with decreased GPC concentrations which continued until hospital discharge (T9). No significant change in lipid concentration was found before treatment and the time of the three-month follow-up (Fig. 3D, F).

### Decreased glycosphingolipid concentration was associated with the conditioning regime

Groups 6 and 4 follow similar expression profiles. A significant decrease in lipid concentrations was observed after the conditioning regime (T5). In Group 6 glycosphingolipids (GSP) were significantly enriched. Decreasing GSP concentrations continued at least until five days after hematopoietic SCR (T8). At the time when patients were discharged from the hospital the concentrations had significantly increased again (T9). At the three-month follow-up no significant differences in GSP concentrations before and after AHSCT could be seen. The expression profiles of two representatives GSP (GlcCer(d18:1/22:0) and GlcCer(d18:1/20:0)) are presented in Fig. 4H-I. No enrichment was found in Group 4, due to too few identified lipids (Fig. 3; Table 1).

**Table 1:** Description of the effects observed in each group of interest from the differential expression analysis of lipidomics data. The table includes the significantly enriched lipid classes (P < .01)

Group	Effect	Lipid classes	*P* value
**G1**	The administration of cyclophosphamide during mobilization (T2) was associated with decreased lipid concentration. Hematopoietic SCR was associated with an increase in some lipid concentrations.	Glycerophosphoinositols (GPI)	2.73e-03
**G2**	Cyclophosphamide administration during mobilization (T2) was associated with a slight decrease in lipid concentrations. During the conditioning regimen (T5), the administration of thymoglobuline and cyclophosphamide was associated with increased lipid concentrations.	Ceramides	4.79e-09
		Glycerophosphoethanolamines (GPE)	2.51e-05
**G3**	G-CSF administration during mobilization (T3) was associated with decreased lipid concentrations. Hematopoietic stem cell collection (T4) was associated with an increase in lipid concentrations, which decreases after reinfusion (T7).	Glycerophosphocholines (GPC)	1.23e-11
**G4**	Thymoglobuline and cyclophosphamide administration during the conditioning regimen (T5) were associated with decreased lipid concentrations.	-	
**G5**	Hematopoietic SCR (T6) was associated with lipid concentration decrease. At the time of the three-month follow-up (T10), lipid concentrations were back to initial concentrations.	Glycerophosphocholines (GPC)	3.96e-08
		Sphingoid bases	2.88e-05
**G6**	The conditioning regimen with cyclophosphamide and thymoglobuline was associated with a more pronounced decrease in lipid concentrations (T5).	Glycosphingolipids (GSP)	8.91e-03

### Disease activity after AHSCT

One year after treatment all 16 patients were in NEDA-3, however, three years after treatment disease activity had occurred for two of the patients (Supplementary Table S1 and Figure S8). For patient 1 evidence of disease activity (EDA) occurred 1.02 years after treatment, while for patient 4 it occurred 2.67 years after treatment. As depicted by Figure S8 the lipidomics changes for these two patients deviated to some degree from the mean lipidomics changes that occurred throughout the treatment, important to note is that both patients deviated from the mean at T1. Further, patient 1 deviated one day after transplantation (T7), while patient 4 deviated both prior to transplantation during mobilization (T2 and T3) and after transplantation (T8 and T10).

At T1 34 features were found to be deviating between patients with EDA and patients with NEDA-3 three years after treatment. Six of these features were identified, out of which three were ceramides. Four lipids had increased concentrations within patients with EDA; Cer(D18:2/23:0), PG(20:3(8Z,11Z,14Z)/18:1(9Z)), C24 ceramide, and PI(18:1(11Z)/16:1(9Z)). While two Cer(D14:!/24:0) and anandamide (20:3,N-3) had decreased concentrations at T1 for patients with EDA within three years after transplantation (Supplementary Figure S9). For all lipids, except Cer(D18:2/23:0), the concentrations did not deviate at T10 between patients with EDA and patients with NEDA-3.

### Newly diagnosed RRMS versus T1 and T10

**Fig. 5:**
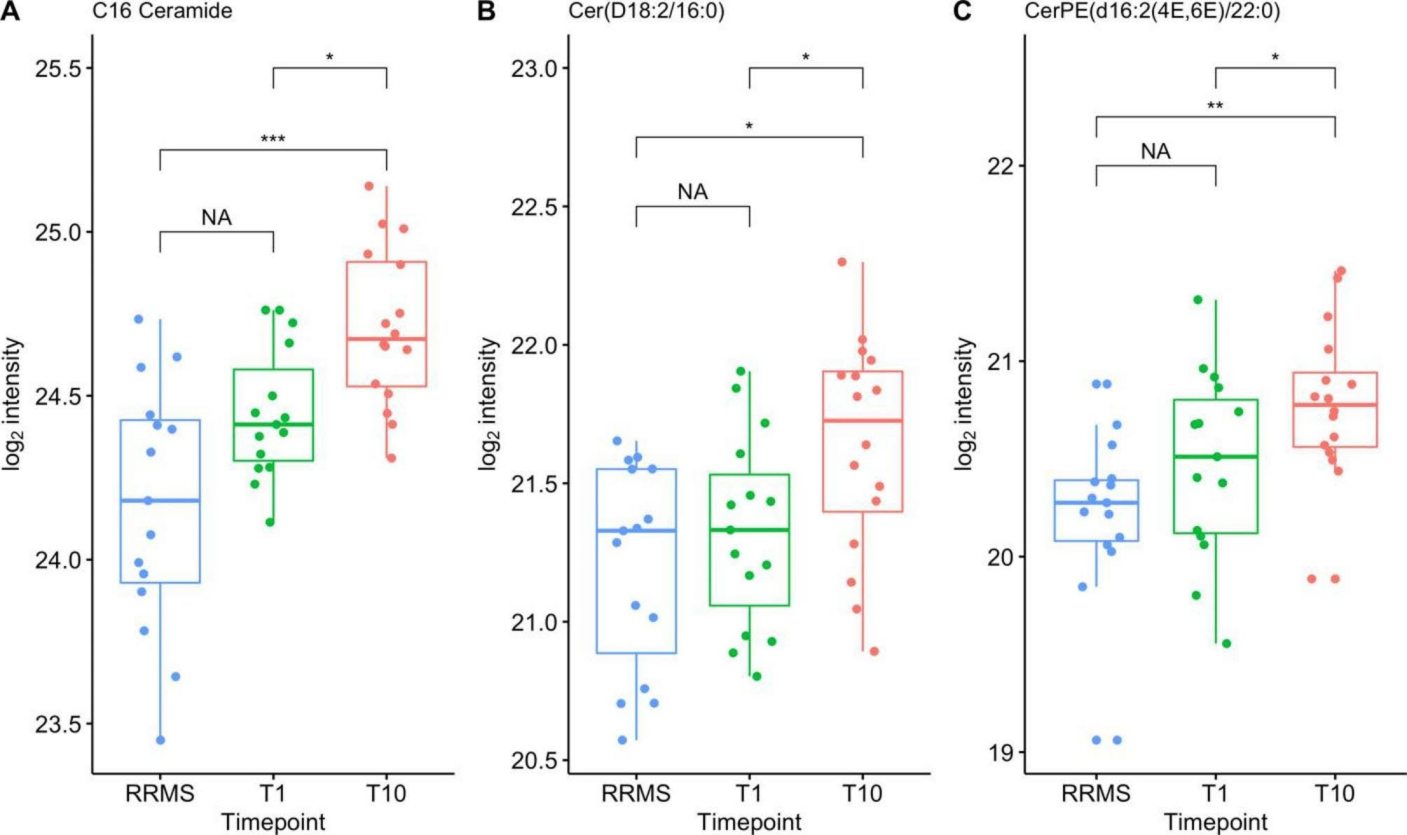
Ceramides with a significant difference in concentration after AHSCT (T10) compared to newly diagnosed RRMS (RRMS in the figure). The significance is illustrated as follows: * P < .05, ** P < .01, and *** P < .001

Fold change analysis on newly diagnosed RRMS versus T1 and T10 for RRMS patients undergoing AHSCT revealed 15 features significantly (*P* < .05), differentiating at T1 and 40 features at T10 (Fig. 3). The features with a significant fold change between T1 and newly diagnosed RRMS, were all increased in concentrations within newly diagnosed RRMS, however, they could not be identified. Out of the 40 features with a significant fold change between T10 and newly diagnosed RRMS, 29 did not have a significant fold change between T1 and newly diagnosed RRMS. Furthermore, all of these 29 features had a positive fold change, indicating that these feature levels were increased at T10 compared to newly diagnosed RRMS. Eight lipids were identified, out of which three were ceramides (Fig. 5); C16 ceramide, Cer(D18:2/16:0), and CerPE(d16:2(4E,6E)/22:0). The other identified lipids were PA(P-18:0/18:1(9Z)), TG(12:0/20:0/20:1(11Z)), FAHFA 17:1/22:1, SM(D16:0/22:0), and GM3 21:1;20/15:0. None of the 31 significant metabolomic differences between newly diagnosed RRMS and T1 and T10 were identified (Supplementary, Figure S2).

### Lipid and metabolite association to clinical measurements

**Fig. 6:**
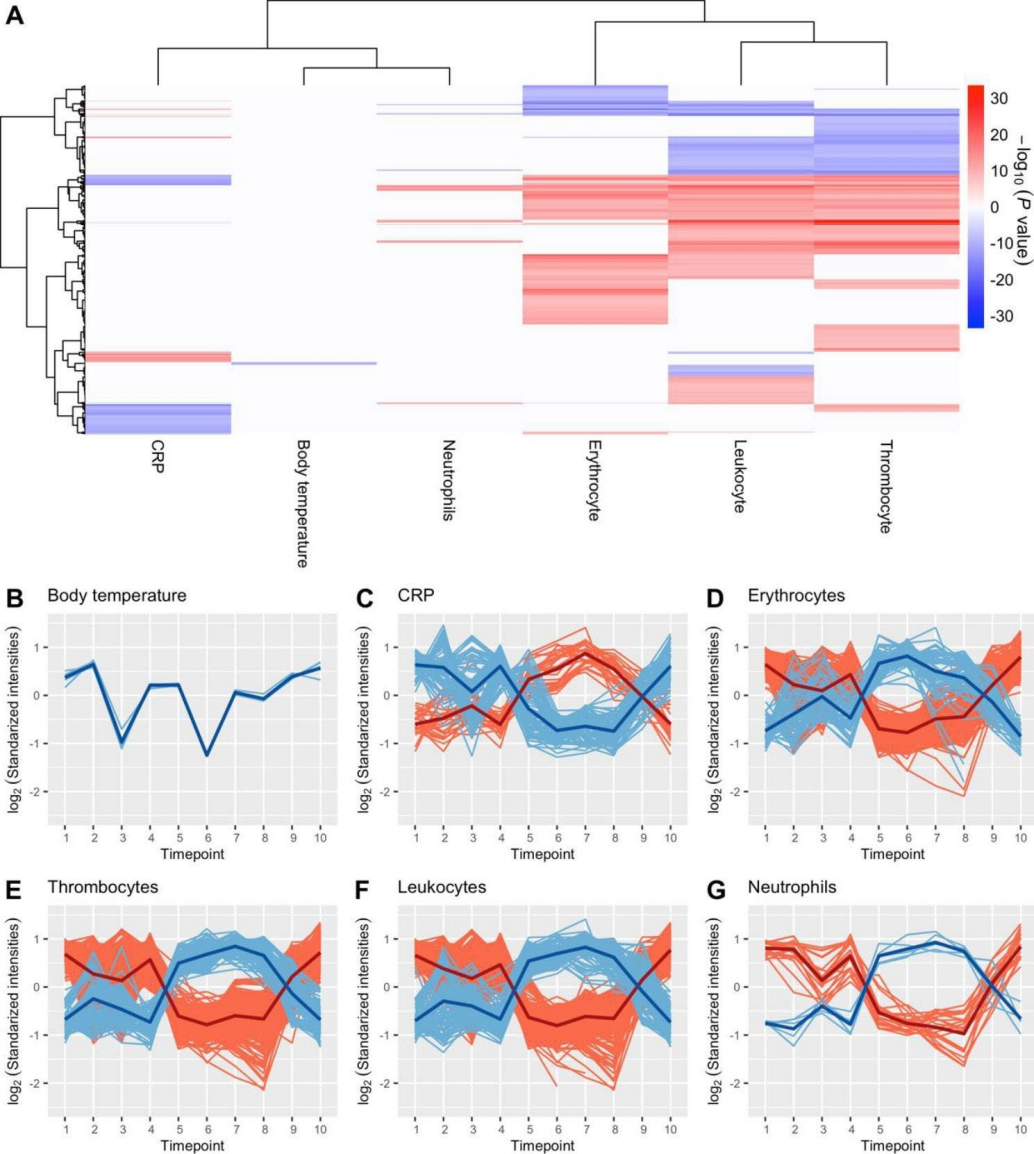
Association profiles of features from the lipidomics dataset associated with one of the six clinical measurements. (A) Heatmap over associated lipids to the six clinical measurements: CRP, body temperature, neutrophil, erythrocyte, leukocyte, and thrombocyte count. (B to G) The expression profiles of the associated features. Blue represents negatively associated features and red positively associated features. A mean curve has been included for each association type

The associations between lipid concentrations to CRP, body temperature, neutrophil, erythrocyte, leukocyte, and thrombocyte count are illustrated in Fig. 6. The clinical measurements expression profile through the treatment is shown in Supplementary Figure S10. Interestingly, 91% (n = 615) of the features were found to be associated with erythrocyte, leukocyte, or thrombocyte counts. Metabolite association to clinical measurement analysis also found the majority of features associated with erythrocyte-, thrombocyte-, and leukocyte count (Supplementary, Figure S11).

Enrichment analysis showed that glycerophosphoethanolamine (GPE) concentrations were mainly associated with erythrocyte counts (Table 2). The concentration of features positively associated with erythrocyte count decreased during both mobilization steps, while it increased during hematopoietic stem cell collection. Further, the conditioning regime was associated with decreased feature concentrations, which continued on until one day after hematopoietic SCR. The opposite expression pattern was found for features negatively associated with erythrocyte count. At the three-month follow-up, feature concentrations were almost back to the initial concentrations (Fig. 6 D).

Features associated with thrombocyte or leukocyte count shared similar expression patterns. For features with a positive association, the mobilization with cyclophosphamide and G-CSF was associated with decreased concentrations. While, for features with a negative association, mobilization with cyclophosphamide was associated with increased concentration, and the administration of G-CSF was associated with decreased analyte concentrations. The conditioning regime was associated with decreased concentrations of positively associated features, which increased again five days after hematopoietic SCR. The opposite pattern was seen for negatively associated features. Feature concentrations were almost back to initial values at the time of the three-month follow-up (Fig. 6 E-F). Enrichment analysis showed that GPC concentrations were mainly associated with thrombocyte counts and ceramide concentrations with leukocyte counts (Table 2). The full enrichment analysis results are presented in Supplements Table S12 to S16.

**Table 2:** Significantly enriched lipid classes (P < .01) associated with the clinical measurements: body temperature, CRP, erythrocytes, thrombocytes, leukocytes, and neutrophil counts

Clinical measurement	Top 3 lipid classes	*P* value
Body Temperature	-	
**CRP**	Glycerophosphocholines (GPC)	3.63e-07
	Glycerophosphoethanolamines (GPE)	4.52e-06
**Erythrocytes**	Glycerophosphoethanolamines (GPE)	1.09e-11
	Ceramides	2.72e-06
	Glycerophosphocholines (GPC)	5.56e-06
	Sphingolipids (SP)	3.50e-04
**Thrombocytes**	Glycerophosphocholines (GPC)	8.13e-09
	Ceramides	2.81e-05
	Glycerophosphoethanolamines (GPE)	8.23e-03
**Leukocytes**	Ceramides	9.30e-10
	Glycerophosphocholines (GPC)	1.41e-06
	Sphingolipids (SP)	4.53e-04
	Fatty amides	6.83e-03
**Neutrophils**	-	

## Discussion

Large and transient changes in protein expression levels associated with the inflammatory milieu in the peripheral blood of RRMS patients undergoing AHSCT has previously been demonstrated but found no long-lasting effects [14]. In this study AHSCT was found to have a more profound impact on lipids in the peripheral blood milieu than metabolites. Lipids are of importance for cell signaling, the formation of the myelin sheath, transport within the CNS [1], and have a central role in immune response and inflammatory processes [36, 37] Differential expression analysis of lipids in RRMS patients undergoing AHSCT demonstrated that a variety of different lipid classes were affected by this treatment. Glycerophosphoinositol concentrations were associated with the administration of cyclophosphamide during mobilization; ceramide, glycerophosphoethanolamine, and glycerosphingolipid concentrations were associated with the conditioning regime where cyclophosphamide and thymoglobuline were administered; and hematopoietic stem cell collection and reinfusion were mainly associated with glycerophosphocholine concentrations. Furthermore, the concentrations of ceramides were strongly associated with leukocyte counts and the two ceramides Cer(d19:1/14:0) and Cer(d20:1/12:0) also showed increased concentrations at the time of the three-month follow-up compared to baseline, suggesting lasting effects of the treatment. The concentration of circulating metabolites was affected by the treatment to a much lesser extent.

One of the features of MS is immune cell infiltration into the CNS, where they release cytokines and inflammatory mediators, promoting inflammation. These immune cells include different types of leukocytes, such as T and B cells, monocytes, and neutrophils [38]. AHSCT is a treatment where hematopoietic stem cells are collected from patients, and after ablation of the immune system, these stem cells are infused into the patient [8–10], with the purpose that a new and more tolerant immune system will be generated [13]. Cyclophosphamide is a drug with immunosuppressive and immunomodulatory properties that in low dosage has been shown to be selective for T cells [39]. Thus, the alterations of lipid and metabolite concentrations during the treatment and their association with leukocytes become of great interest to further understand AHSCT effect on the peripheral blood milieu of RRMS patients.

Here, a significant decrease in GPI concentrations was found to be associated with the administration of cyclophosphamide during mobilization. It is previously known that GPIs play a role in inflammatory and immune responses; more specifically, they act as modulators of T-cell activity [40, 41]. During the conditioning regimen, a higher dose of cyclophosphamide in combination with thymoglobuline is administered to deplete immune cells, however, no significant change in GPI concentration occurred. This is surprising since thymoglobuline is a polyclonal T-cell depleting antibody targeting several T- and B-cell antigens [42] it would be expected to affect GPI concentrations. Thus, GPI concentrations are more likely linked to the peripheral blood milieu and the mobilization of hematopoietic stem cells, rather than the number of T cells.

Ceramides and GPE concentrations significantly increased in response to the conditioning regime with cyclophosphamide and thymoglobuline. Since mobilization with cyclophosphamide had no effect on these lipid concentrations, the effects seen are likely to be associated with the administration of thymoglobuline. Ceramides, a subclass of SP, are particularly abundant in myelin [1, 43–45], and expressed by multiple immune cells such as neutrophils, T- and B-cells [46]. Disturbances in SP signaling are involved in various neuroinflammatory or neurodegenerative diseases and strongly affect inflammation in autoimmune diseases [1]. Previous studies on cerebrospinal fluid (CSF) have shown increased Cer(d18:1/16:0) concentrations in MS patients compared to healthy controls [47], further alterations in SP pathways, particularly ceramide concentration, play an important role in oligodendrocyte damage and acute demyelination [1]. Thus, suggesting that these could potentially have an important role in disease pathology.

Further, the concentrations of ceramides were found to be associated with leukocyte counts. Previous studies have found altered ceramide levels within leukocytes, which may account for the changes seen in ceramide concentrations in MS patients [5]. Further, leukocyte count, which is related to the innate immune system, has been shown to on average be elevated in MS patients compared to healthy controls [48]. It has been suggested that the platelet-leukocyte interaction affects the pathophysiology of MS and plays a central role in initiating increased infiltration of autoreactive T cells, thus forming new neuroinflammatory lesions in the CNS [49]. Interestingly the two ceramides Cer(d19:1/14:0) and Cer(d20:1/12:0) were among the few lipids that showed increased levels in the three months follow-up, compared to before treatment. At this time no previous studies have identified the role of these two ceramides in AHSCT and MS. Further analysis is needed to determine their role in the improvement seen in RRMS patients undergoing AHSCT.

GSP concentration levels decreased in response to the conditioning regime with cyclophosphamide and thymoglobuline, while lipid concentrations were not affected by the mobilization. Since GSPs are expressed by various immune cells such as neutrophils, T and B cells [46], this decrease in lipid concentrations could be a direct response to the decreased amount of T cells and other immune cells. However, it is important to consider the difficulties in distinguishing between the effects of thymoglobuline and cyclophosphamide administration.

GPC was found to be enriched in association with leukocytes, thrombocytes, and erythrocytes, as well as differentially expressed through the treatment. GPC represents a class of lipids formed during phosphatidylcholine breakdown, a membrane phospholipid also present in myelin. Since other anabolic routes cannot form GPCs, it is a specific indicator of membrane breakdown [50, 51]. Mobilization with G-CSF moves CD34 + stem cells from the bone marrow into the peripheral blood [9], which was associated with increased GPC concentration. Hematopoietic SCR was however associated with decreased analyte levels. Thus, GPC concentrations were affected by the presence of CD34 + stem cells. However, its exact role in RRMS patients undergoing AHSCT is unknown.

A comparison of the differentially expressed features between newly diagnosed RRMS patients and T1 and T10 showed that the ceramides C16 ceramide, Cer(D18:2/16:0), and CerPE(d16:2(4E,6E)/22:0) were significantly increased in concentration after transplantation. These ceramides did however not significantly differ in concentration between newly diagnosed RRMS and T1, thus this increase in ceramide concentrations is a result of the treatment and not the disease itself.

Finally, to investigate if any lipids could indicate clinical relevance regarding treatment outcome lipid concentrations before treatment were compared between patients with EDA within three years after transplantations and patients with NEDA-3. The ceramides Cer(D18:2/23:0), C24 ceramide, and Cer(D14:1/24:0) were found to differ in concentration between patients with EDA and patients with NEDA-3. Thus, further underlying the importance of ceramides within the disease as well as AHSCT. However, it is important to note that only two patients had EDA, while 14 patients were in NEDA-3. Further analysis with a larger cohort is needed to ensure statistical significance regarding the potential biomarkers found.

### Study strengths and limitations

Lipids are one of the most extensive and heterogenous groups of molecules present in the human body. It is estimated that more than the entire human lipid metabolism contains more than 100,000 lipid species. Lipids play a crucial role in signaling, energy metabolism, and forming the structure of cell membranes, making them essential for cellular functioning. Today, more than 47,000 lipid species are available in LipidMaps database [28], which is the world’s most extensive public lipid-only database. Lipidomics technologies using e.g. LC-HRMS allows for the analysis of several thousand lipid species in human biofluids, thus opening up for novel findings. However, this also comes with one of the major challenges to the field; correct identification of lipids. Here, in-house compound libraries and state-of-the-art in silico methodologies were used to identify lipids, but still, this does not enable us to with high confidence identify all features, which is a limitation of the study. Another limitation is the relatively low number of individuals included in the study. However, similar responses were seen across patients, which likely is associated with effects of the treatment, resulting in limited variability across individuals. Another limitation is the lack of healthy controls, which would have eased the interpretation of long-term effects, in relation to the effects of the treatment. This also opens up for investigating these patients over a longer time period, in connection to disease progression and healthy controls and long-term alterations in relation to lipid species. Finally, the small number of patients with EDA within three years after treatment prevents the identification of statistically significant biomarkers for patients’ clinical outcome from AHSCT.

## Conclusions

Significant alterations of lipid concentrations relating to specific classes were found throughout the course of AHSCT. In the vast majority of these lipids, the concentrations were back to pre-transplant levels at three-months follow-up. Thus, the variation in lipid concentration over the course of AHSCT reflects on the transient changes in the peripheral blood milieu, during the treatment, rather than the immunological alterations assumed to be the cause of clinical improvement within RRMS patients treated with AHSCT. For two ceramides significantly increased concentration after AHSCT compared to baseline were noted, suggesting a long-term effect.

Previous studies have found changes in the levels of ceramides among MS patients compared to healthy controls. These alternations were linked with physical disability and neurodegeneration of the retina. High levels of Cer16:0 was associated with increased odds of expanded disability status scale worsening, while Hex-Cer22:0 and DH-HexCer22:0 were correlated with increased rates of neurodegeneration [5]. Another study found increased levels of anti-Cer IgGs in serum of MS patients compared to healthy controls [52]. Here, Cer(d19:1/14:0) and Cer(d20:1/12:0) were found to be increased in concentration at the three-month follow-up compared to before treatment. In addition, C16 ceramide, Cer(D18:2/16:0), and CerPE(d16:2(4E,6E)/22:0) were found to be significantly increased in concentration after AHSCT compared to prior to treatment as well as to newly diagnosed RRMS patients. Thus, suggesting that circulating ceramides are strongly affected by AHSCT. The knowledge of variations in GPIs, ceramides, GPEs, GSPs, and GPCs through AHSCT can contribute to a better understanding of the short-term impact of the treatment as well as for the underlying mechanism of the improvement seen in RRMS patients. Such effects on the lipidome may also apply to AHSCT treatment for other medical conditions. Finally, comparisons between different MS treatments and the correlation to clinical outcome would help to better understand these findings as well as the potential role of ceramides as biomarkers for predicting clinical outcome.

## Electronic supplementary material

Below is the link to the electronic supplementary material.

Supplementary Material 1

## Data Availability

The datasets used and analyzed during the current study are available from the corresponding author on reasonable request.

## Abbreviations

AHSCT: Autologous hematopoietic stem cell transplantation
ANOVA: Analysis of variance
AU: Approximately Unbiased
CNS: Central nervous system
CSF: Cerebrospinal fluid
CRP: C-reactive protein
CV: Coefficient of variation
EDA: Evidence of Disease Activity
EDSS: Expanded Disability Status Scale
EMMs: Estimated marginal means
FDR: False discovery rate
G-CSF: Granulocyte colony stimulating factor
GPC: Glycerophosphocholine
GPE: Glycerophosphoethanolamine
GPI: Glycerophosphoinositol
GSP: Glycosphingolipids
HPLC: High-performance liquid chromatography
HRMS: Liquid chromatography high-resolution mass spectrometry
LC-HRMS: Liquid chromatography high-resolution mass spectrometry
MS: Multiple sclerosis
NEDA-3: No evidence of disease activity
PCA: Principal component analysis
QC: Quality control
RRMS: Relapsing-remitting multiple sclerosis
SCR: Stem cell reinfusion
SP: Sphingolipids
SPMS: secondary-progressive multiple sclerosis
