# Supplementary material for: Autologous hematopoietic stem cell transplantation significantly alters circulating ceramides in peripheral blood of relapsing-remitting multiple sclerosis patients
Source: Lipids Health Dis. 2023 Jul 7;22:97. doi: 10.1186/s12944-023-01863-7 (PMC10327322; doi:10.1186/s12944-023-01863-7)
Supplement: Supplementary file 1 — Supplementary Material 1 [file 12944_2023_1863_MOESM1_ESM.docx]

## Supplementary Information

- **Table S1.** Patient clinical data after treatment.
- **Table ~~S1~~ S2**. List of internal standards used in the precipitation solution for metabolite extraction.
- **Table ~~S2~~ S3**. List of internal standards used in the precipitation solution for lipid extraction.
- **Table ~~S3~~ S4**. Parameters used for Feature Finder.
- **Table ~~S4~~ S5**. Parameters used for Feature Linker.
- **Table ~~S5~~ S6**. Parameters used for in silico identification using CSIFingerID.
- **Figure S1**. Post mass calibration of identified lipids.
- **Figure S2**. Differential expression analysis of metabolomics data.
- **Table ~~S6~~ S7**. Results from enrichment analysis of differentially expressed lipids within Group 1.
- **Figure S3**. Boxplots of identified lipids within enriched lipid classes of Group 1.
- **Table ~~S7~~ S8**. Results from enrichment analysis of differentially expressed lipids within Group 2.
- **Figure S4**. Boxplots of identified lipids within enriched lipid classes of Group 2.
- **Table ~~S8~~ S9**. Results from enrichment analysis of differentially expressed lipids within Group 3.
- **Figure S5**. Boxplots of identified lipids within enriched lipid classes of Group 3.
- **Table ~~S9~~ S10**. Results from enrichment analysis of differentially expressed lipids within Group 5.
- **Figure S6**. Boxplots of identified lipids within enriched lipid classes of Group 5.
- **Table ~~S10~~ S11**. Results from enrichment analysis of differentially expressed lipids within Group 6.
- **Figure S7**. Boxplots of identified lipids within enriched lipid classes of Group 6.
- **Figure S8.** PCA for patients with EDA within three years after AHSCT.
- **Figure S9.** Lipid levels prior to and after AHSCT for lipids deviating between patients with EDA within three years after treatment and patients with NEDA-3 after three years.
- **Figure ~~S8~~** **S10**. Expression profiles of the six clinical measurements analyzed during AHSCT.
- **Figure ~~S9~~** **S11**. Metabolite association to clinical measurements through AHSCT.
- **Table ~~S11~~ S12**. Results from enrichment analysis of lipids associated with C-reactive protein (CRP).
- **Table ~~S12~~ S13**. Results from enrichment analysis of lipids associated with erythrocyte counts.
- **Table ~~S13~~ S14**. Results from enrichment analysis of lipids associated with thrombocyte counts.
- **Table ~~S14~~ S15**. Results from enrichment analysis of lipids associated with leukocyte counts.
- **Table ~~S15~~ S16**. Results from enrichment analysis of lipids associated with neutrophil counts.

**Table S1.** Patient clinical data before and after undergoing AHSCT including Expanded Disability Status Scale (EDSS), relapses, MRI activity, and No Evidence of Disease Activity (NEDA-3) according to the following parameter; confirmed disease worsening, clinical relapses, and MRI events.

| **Patient nr** | **EDSS** | | | | | **Relapses** | | **MRI activity** | | **NEDA-3** | |
| --- | --- | --- | --- | --- | --- | --- | --- | --- | --- | --- | --- |
|  | **Baseline** | **1 y** | **2 y** | **3 y** | **Censored y** | **Relapses** | **Censored y** | **MRI activity** | **Censored y** | **Progression** | **Censored y** |
| 1 | 4 | 3.5 | 3.5 | 3.5 | 4.21 | 1 | 1.43 | 1 | 1.02 | 1 | 1.02 |
| 2 | 3.5 | 2 | 1 | 1 | 6.04 | 0 | 6.04 | 0 | 6.04 | 0 | 6.04 |
| 3 | 3.5 | 3.5 | 3 | 3 | 5.47 | 0 | 5.47 | 0 | 5.64 | 0 | 5.47 |
| 4 | 3.5 | 3 | 3 | 3 | 5.31 | 1 | 2.67 | 0 | 5.31 | 1 | 2.67 |
| 5 | 4 | 2 | 2 | NA | 5.37 | 0 | 5.37 | 0 | 5.37 | 0 | 5.37 |
| 6 | 3.5 | 3.5 | NA | 3.5 | 4.95 | 0 | 4.95 | 0 | 3.98 | 0 | 3.98 |
| 7 | 2.5 | 3 | NA | 3 | 6.32 | 0 | 6.32 | 0 | 6.48 | 0 | 6.32 |
| 8 | 2 | 0 | 0 | 0 | 7.04 | 0 | 7.04 | 1 | 6.98 | 1 | 6.98 |
| 9 | 2 | 0 | 0 | 0 | 6.11 | 0 | 6.11 | 0 | 6.11 | 0 | 6.11 |
| 10 | 4 | 2 | 1.5 | 1.5 | 6.63 | 1 | 3.43 | 1 | 4.18 | 1 | 3.43 |
| 11 | 4 | 3.5 | 3.5 | 3 | 5.18 | 1 | 3.88 | 0 | 5.18 | 1 | 3.88 |
| 12 | 3 | 1.5 | 0 | 0 | 5.23 | 0 | 5.23 | 1 | 3.01 | 1 | 3.01 |
| 13 | 3.5 | 3.5 | 2 | 2 | 5.05 | 0 | 5.05 | 0 | 5.05 | 0 | 5.05 |
| 14 | 1.5 | 0 | 0 | 0 | 6.22 | 0 | 6.22 | 0 | 3.99 | 0 | 3.99 |
| 15 | 6 | 3 | 4.5 | 4.5 | 6.11 | 0 | 6.11 | 0 | 5.98 | 0 | 5.98 |
| 16 | 2 | 1 | 0 | NA | 3.49 | 0 | 3.49 | 1 | 3.49 | 1 | 3.49 |

###

**Table ~~S1~~ S2.** List of the internal standards used in the precipitation solution for metabolite extraction with molecular formulas, detected m/z, retention time, and concentration in precipitation solution.

| **Internal standard** | **Molecular Formula** | **m/z** | **Rt (min)** | **Conc (pg/µL)** |
| --- | --- | --- | --- | --- |
| Amfetamin - d6 | C9D6H7N | 142.1497 | 4.2 | 2 |
| 7-Aminonitrazepam -d5 | C15H8D5N3O | 257.1445 | 4.91 | 2 |
| MDA - d5 | C10D5H8NO2 | 185.1333 | 5.2 | 2 |
| Metamfetamin - d5 | C10D5H10N | 155.1591 | 5.24 | 2 |
| Kodein -d3 | C18H18D3NO3 | 303.1783 | 5.26 | 2 |
| Noroxikodon -d3 | C17H16D3NO4 | 305.1575 | 5.35 | 2 |
| MDMA [Ecstasy] -d5 | C11D5H10NO2 | 199.1489 | 5.56 | 2 |
| 6-Monoacetylmorfin [6-AM] -d3 | C19D3H18NO4 | 331.1732 | 5.81 | 2 |
| MDEA -d5 | C12D5H12NO2 | 213.1646 | 6.05 | 2 |
| Etylmorfin -d5 | C19D5H18NO3 | 319.2065 | 6.15 | 2 |
| Oxikodon -d3 | C18H18NO4D3 | 319.1732 | 6.15 | 2 |
| 7-Aminoklonazepam -d4 | C15D4H8ClN3O | 290.0993 | 6.27 | 2 |
| Bensoylekgonin -d3 | C16D3H16NO4 | 293.1575 | 6.53 | 2 |
| Hydromorfon -d3 | C17H16NO3D3 | 289.1626 | 6.64 | 2 |
| Morfin -d3 | C17H16NO3D3 | 289.1626 | 6.64 | 2 |
| Tramadol 13C,D3 | 13CC15D3H22NO2 | 268.2185 |  | 2 |
| 7-Aminoflunitrazepam -d7 | C16D7H7FN3O | 291.1633 | 6.87 | 2 |
| Metylfenidat -d9 | C15H14NO2D9 | 259.2367 | 6.88 | 2 |
| alfa-Hydroximidazolam -d4 | C18D4H9ClFN3O | 346.1055 | 8.16 | 2 |
| Fentanyl -d5 | C22D5H23N2O | 342.2588 | 8.19 | 2 |
| Buprenorfin -d4 | C29H37NO4D4 | 472.3359 | 8.5 | 2 |
| Metadonmetabolit EDDP -d3 | C20D3H2ON | 281.2097 | 8.5 | 2 |
| alfa-Hydroxialprazolam -d5 | C17D5H8ClN4O | 330.1165 | 8.9 | 2 |
| Oxazepam -d5 | C15D5H6ClN2O2 | 292.0896 | 8.9 | 2 |
| alfa-Hydroxitriazolam -d4 | C17D4H8Cl2N4O | 363.0712 | 8.94 | 2 |
| Desmetyldiazepam -d5 | C15D5H6ClN2O | 276.0947 | 9.07 | 2 |
| Lorazepam -d4 | C15D4H6Cl2N2O2 | 325.0443 | 9.07 | 2 |
| Metadon -d3 | C21D3H24NO | 313.2354 | 9.14 | 2 |
| Temazepam -d5 | C16D5H8ClN2O2 | 306.1052 | 9.4 | 2 |
| THC-COOH -d3 | C21H25O4D3 | 348.2249 | 11.82 | 2 |
| TMAO-A2:E31d3 (Trimethylamine N-oxide-d3) | (CD3)3N(O) | 85.1322 | 0.58 | 6 |
| Noradrenalin-d6 (Norepinephrine-d6) | C8H5D6NO3 (water loss: C8H3D6NO2) | 158.1088 | 0.63 | 6 |
| Normetadrenalin-d3 (Normetanephrine-d3) | C9H10D3NO3 (water loss: C9H8D3NO2) | 169.1056 | 0.64 | 6 |
| Metadrenalin-d3 | C10D3H12NO3 | 201.1313 | 0.7 | 6 |
| 3-metoxytyramin-d4 | C9H9D4NO2 | 172.1270 | 1.1 | 6 |
| 5-HIAA-D5 (Hydroxyindole acetic acid -d5) | C10H4D5NO3 | 197.0969 | 4.18 | 6 |
| HVA-d5 (Homovanillic acid - d5) | C9H5D5O4 | 188.0966 | 4.2 | 6 |
| VMA-D3 (Vanilmandelic acid -d3) | C9H7D3O5 | 202.0789 | 4.2 | 6 |
| Cortisone-d8 | C21D8H20O5 | 369.2512 | 8.41 | 6 |
| Cortisol-d4 | C21H26D4O5 | 367.2417 | 8.43 | 6 |
| 21-deoxycortisol-d8 | C21H22O4D8 | 355.2719 | 8.99 | 6 |
| Androstendione-d7 | C19H19D7O2 | 294.2445 | 10.02 | 6 |
| 17-alfa-hydroxyprogesterone-d8 | C21H22O3D8 | 339.2770 | 10.16 | 6 |
| Levonorgestrel-d6 | C21H22D6O2 | 319.2539 | 10.41 | 6 |
| Progesteron-d9 | C21H21O2D9 | 324.2884 | 11.23 | 6 |

**Table ~~S2~~ S3.** List of the internal standards used in the precipitation solution for lipid extraction with molecular formulas, detected m/z, retention time, and concentration in precipitation solution, as well as the polarity and detected adduct used for optimal detection.

| **Internal standard** | **Polarity** | **Molecular Formula** | **m/z** | **Rt (min)** | **Detected adduct** | **Conc (pg/µL)** |
| --- | --- | --- | --- | --- | --- | --- |
| 17-α-Hydroxyprogesterone-d8 | Pos | C21H22O3D8 | 339.2770 | 1.35 | +H | 36 |
| Androstendione-d7 | Pos | C19H19D7O2 | 294.2445 | 1.16 | +H | 36 |
| Cholesterol (d7) | Pos | C27H37D7 | 376.3955 | 4.45 | -H2O | 667 |
| Cholesterol Ester 18:1(d7) | Pos | C45H71D7O2 | 675.6779 | 10.12 | +NH4 | 2333 |
| DG 15:0-18:1(d7) | Pos | C36H61D7O5NH3 | 605.5844 | 5.33 | +NH4 | 67 |
| Levonorgestrel-d6 | Pos | C21H22D6O2 | 319.2539 | 1.47 | +H | 36 |
| LPC 18:1(d7) | Pos | C26H45D7NO7P | 529.3994 | 3.13 | +H | 167 |
| LPE 18:1(d7) | Pos | C23H39D7NO7P | 487.3524 | 3.13 | +H | 33 |
| MG 18:1(d7) | Pos | C21H33D7O4 | 364.3439 | 3.41 | +H | 13 |
| PC 15:0-18:1(d7) | Pos | C41H73D7NO8P | 753.6134 | 4.55 | +H | 1067 |
| PE 15:0-18:1(d7) | Pos | C38H67D7NO8P | 711.5664 | 4.59 | +H | 33 |
| Progesteron-d9 | Pos | C21H21O2D9 | 324.2884 | 1.95 | +H | 36 |
| SM 18:1(d9) | Pos | C41H72D9N2O6P | 738.647 | 4.36 | +H | 200 |
| TG 15:0-18:1(d7)-15:0 | Pos | C51H89D7O6NH3 | 829.7985 | 9.06 | +NH4 | 367 |
| THC-COOH-d3 | Pos | C21H25O4D3 | 348.2249 | 2.66 | +H | 36 |
| LPE 18:1(d7) | Neg | C23H39D7NO7P | 485.3378 | 3.13 | -H | 33 |
| PE 15:0-18:1(d7) | Neg | C38H67D7NO8P | 709.5519 | 4.61 | -H | 33 |
| PG 15:0-18:1(d7) | Neg | C39H68D7O10P | 740.5464 | 6.53 | -H | 200 |
| Phosphatidylethanol-d5 | Neg | C39H70D5O8P | 706.5441 | 7.76 | -H | 97 |
| PI 15:0-18:1(d7) | Neg | C42H72D7O13P | 828.5625 | 6.36 | -H | 67 |

**Table ~~S3~~ S4.** Parameters used for Feature Finder.

| **Parameter** | **Lipidomics** | **Metabolomics** |
| --- | --- | --- |
| **Common:** | | |
| Noise threshold int | 40 000 | 500 |
| Chrom peak snr | 5 | 2 |
| Chrom fwhm | 5 | 3 |
| **mtd** | | |
| Mass error ppm | 3 | 5 |
| Reestimate mt sd | True | True |
| Quant method | Area | Area |
| Trace termination criterion | Outlier | Outlier |
| Trace termination outliers | 5 | 5 |
| Min sample rate | 0.5 | 0.5 |
| Min trace length | 5 | 3 |
| Max trace length | -1 | -1 |
| **edp** | | |
| enabled | True | True |
| With filtering | Auto | Auto |
| Min fwhm | 3 | 3 |
| Max fwhm | 60 | 60 |
| Mass trace snr filtering | False | False |
| **ffm** | | |
| Local rt range | 10 | 10 |
| Local mz range | 6.5 | 6.5 |
| Charge lower bound | 1 | 1 |
| Charge upper bound | 1 | 3 |
| Report summed ints | False | False |
| Enable RT filteringe | True | True |
| Isotope filtering model | 5% | 5% |
| Mz scoring 13C | True | False |
| Use smoothed intensites | False | True |
| Report convex hulls | True | True |

**Table ~~S4~~ S5.** Parameters used for Feature Linker.

| **Parameter** | **Lipidomics** | **Metabolomics** |
| --- | --- | --- |
| Keep subelements | False | False |
| threds | 20 | 20 |
| **algorithm** | | |
| Use identification | False | False |
| Nr partitions | 100 | 100 |
| Ignore charge | True | True |
| Ignore adducts | True | True |
| **distance_RT** | | |
| Max difference | 7.0 | 7.0 |
| **distance_MZ** | | |
| Max difference | 7.0 | 7.0 |
| unit | ppm | ppm |

###

###

**Table ~~S5~~ S6.** Parameters used for in silico identification using CSIFingerID.

| **Parameters** | **Positive mode** | **Targeted** | |
| --- | --- | --- | --- |
|  |  | **Positive mode** | **Negative mode** |
| SIRIUS | | | |
| Intrument | Orbitrap | Orbitrap | Orbitrap |
| Filter by isotope pattern | True | True | True |
| MS/MS isotope score | Ignore | Ignore | Ignore |
| MS2 MassDev (ppm) | 20.0 | 20.0 | 20.0 |
| Candidates | 10 | 10 | 10 |
| Candidates per ion | 1 | 1 | 1 |
| Consider only formulas in DBs | All | All | All |
| Possible ion | [M+H]+,[M+Na]+,[M-H2O+H]+,[M+H3N+H]+,[M-H4O2+H]+,[M+K]+ | [M+H]+,[M+Na]+,[M-H2O+H]+,[M+H3N+H]+,[M-H4O2+H]+,[M+K]+ | M-H2O-H]-,[M+Cl]-,[M+Br]-,[M-H]- |
| Tree timeout | 0 | 0 | 0 |
| Compound timeout | 0 | 0 | 0 |
| Use heuristic above m/z | 600 | 300 | 300 |
| Use heuristic only above m/z | 600 | 650 | 650 |
| CSI:FingerID | | | |
| Search in DBs | All | All | All |
| Fallback adducts | [[M+C2H3N+H]+,[M+C2H6OS+H]+,[M-H+Na+Na]+,[M+C2H3N+Na]+,[M+C3H8O+H]+,[M]+,[M+H2O+H]+,[M+K]+,[M+CH4O+H]+,[M+C4H6N2+H]+,[M+H]+,[M-H+K+K]+,[M-H2O+H]+,[M+Na]+,[M+H3N+H]+] | [[M+C2H3N+H]+,[M+C2H6OS+H]+,[M-H+Na+Na]+,[M+C2H3N+Na]+,[M+C3H8O+H]+,[M]+,[M+H2O+H]+,[M+K]+,[M+CH4O+H]+,[M+C4H6N2+H]+,[M+H]+,[M-H+K+K]+,[M-H2O+H]+,[M+Na]+,[M+H3N+H]+] | [M+C2HF3O2-H]-,[M-H2O-H]-,[M+C2H4O2-H]-,[M+Cl]-,[M+C2H3N-H]-,[M+Br]-,[M+CH2O2-H]-,[M-H]-,[M]-,[M-H+K-H]-,[M-H+Na-H]- |
| Formula score threshold | True | True | True |
| Canapous | | | |
| Canapous | True | True | True |


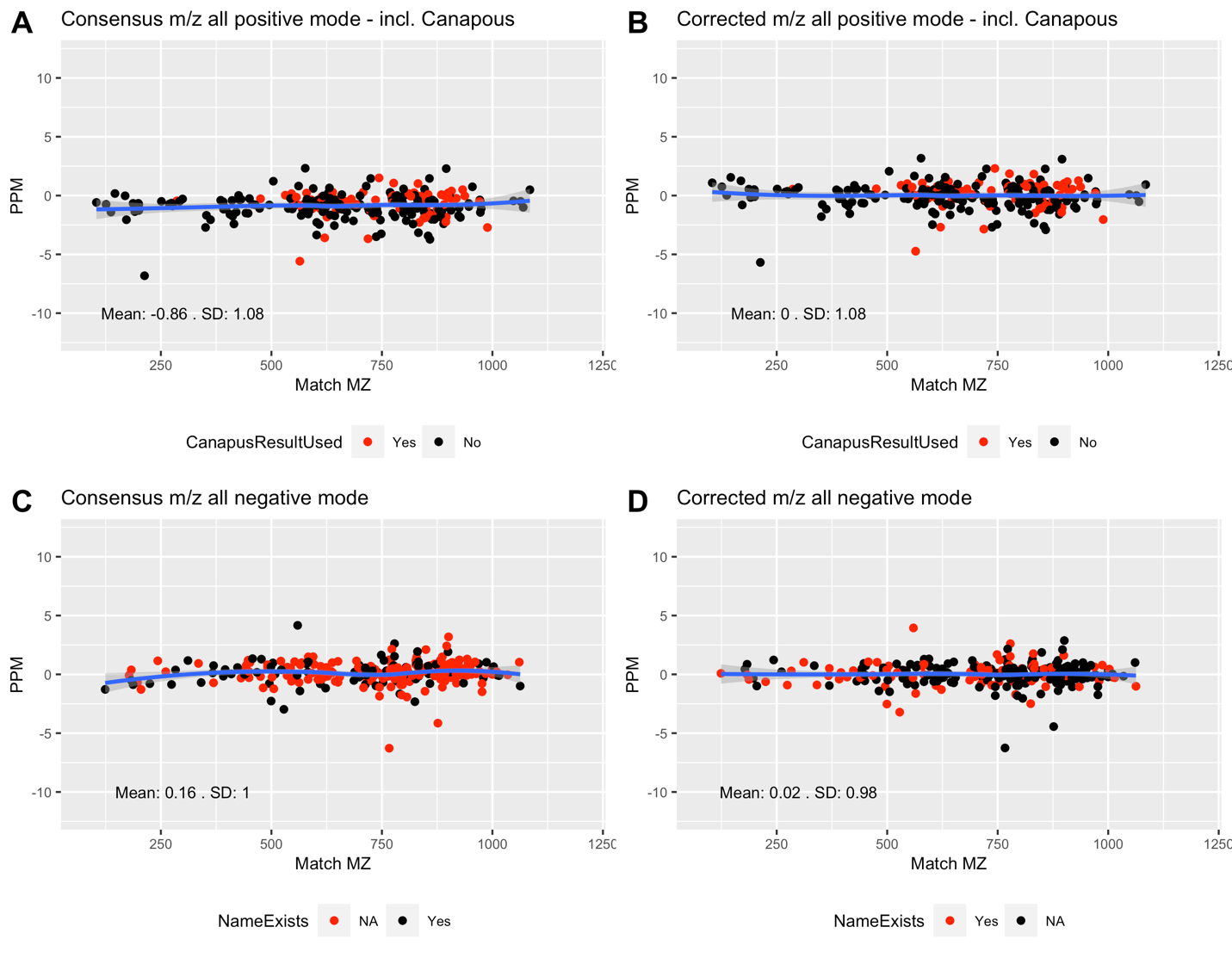


**Figure S1.** Post mass calibration of identified lipids. The consensus m/z versus ppm error to identified feature as well as after post-mass recalibration with LOESS. (A and B) Positive mode includes matches from Canapous as well. (C and D) Features detected in negative mode.


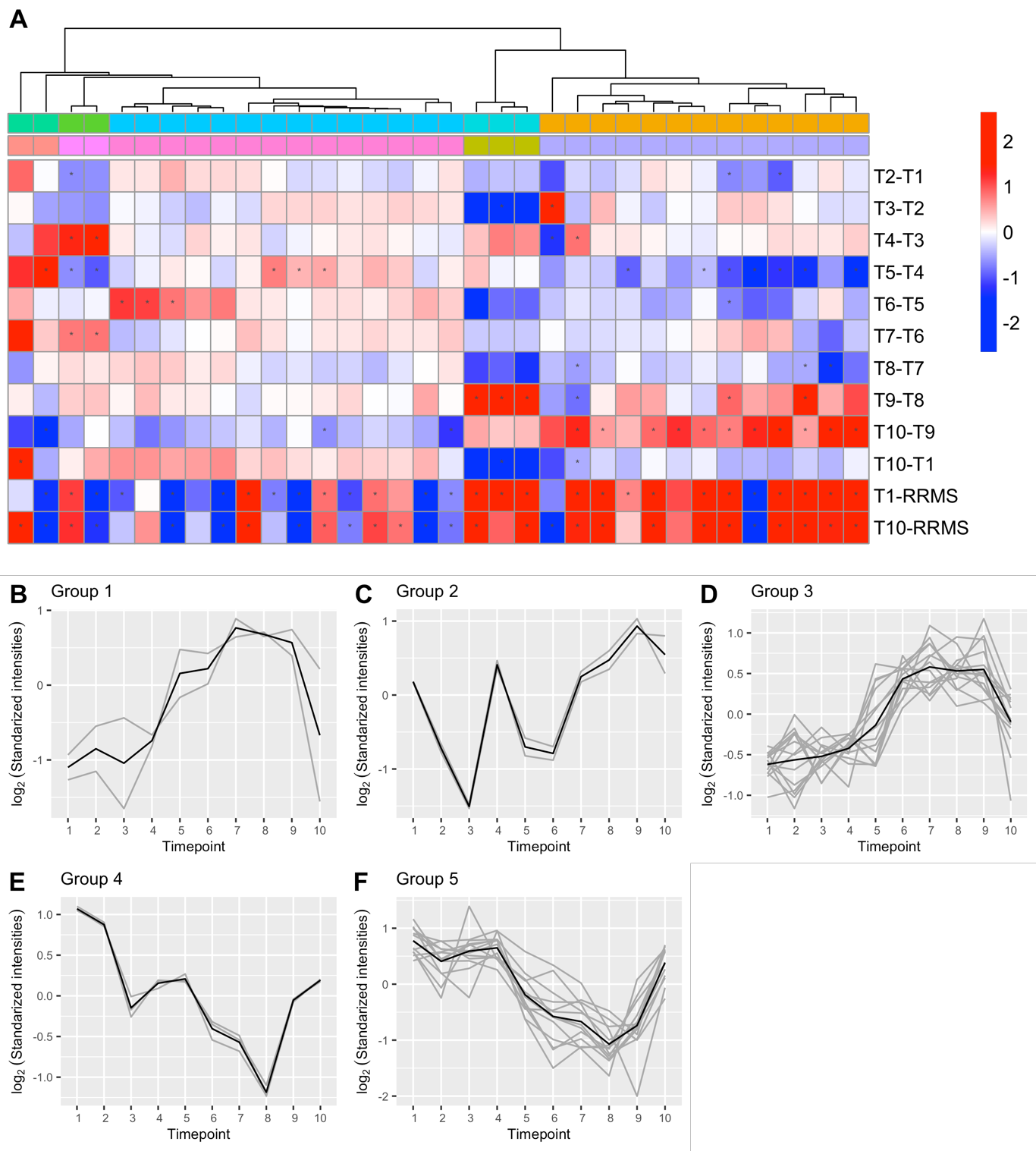


**Figure S2.** Differential expression analysis of metabolomic data. (A) Heatmap over the log_2_(fold change) for adjacent sampling time points during AHSCT for RRMS patients' metabolomics data, including the log_2_(fold change) for the three-months follow-up (T10) and baseline (T1) comparison. In addition, newly diagnosed RRMS (RRMS in the figure) have been compared to t1 and T10. The red gradient indicates a positive log_2_(fold change) and the blue indicates a negative log_2_(fold change). Significant (*P* < .05) log_2_(fold change) is indicated with an asterisk. (B to F) The expression profiles of the five groups identified in the heatmap.

**Table ~~S6~~ S7.** Results from enrichment analysis of identified differentially expressed lipids within Group 1.

| **Lipid class** | **Hits / Total** | **Expected** | **Raw *P* value** | **Holm *P* value** | **FDR** | **Lipids** |
| --- | --- | --- | --- | --- | --- | --- |
| Glycerophospho-  inositols | 4 / 4360 | 0.593 | 0.00273 | 1 | 1 | PI(16:0/18:1(9Z)); PI(16:0/22:6(4Z,7Z,10Z,13Z,16Z,19Z)); PI(18:1(9Z)/20:4(5Z,8Z,11Z,14Z)); PI(16:0/20:4(5Z,8Z,11Z,14Z)) |
| Diradylglycerols | 3 / 3660 | 0.498 | 0.0132 | 1 | 1 | DG(16:0/18:1(9Z)/0:0); DG(18:0/16:0/0:0); DG(20:0/18:0/0:0) |
| Glycerophospho-  cholines | 3 / 4700 | 0.64 | 0.0255 | 1 | 1 | PC(12:0/13:0); PC(10:0/18:0); PC(13:0/0:0) |
| Bile acids | 1 / 311 | 0.0423 | 0.0415 | 1 | 1 | Ursodeoxycholic acid |
| Sphingolipids | 1 / 487 | 0.0663 | 0.0642 | 1 | 1 | Glucosylceramide (d18:1/12:0) |
| Glycosyldiradyl-  glycerols | 2 / 3500 | 0.476 | 0.0815 | 1 | 1 | MGDG(10:0_10:0); MGDG(18:3(9Z,12Z,15Z)/18:3(9Z,12Z,15Z)) |
| Glycerophospho-  serines | 2 / 4140 | 0.564 | 0.109 | 1 | 1 | PS(20:1(11Z)/22:0); PS(46:4) |
| Glycerophospho-  glycerols | 1 / 4190 | 0.57 | 0.438 | 1 | 1 | PG(20:3(8Z,11Z,14Z)/18:1(9Z)) |
| Phosphosphingo-  lipids | 1 / 4800 | 0.653 | 0.483 | 1 | 1 | SM(d18:1/18:0) |
| Ceramides | 1 / 5560 | 0.756 | 0.536 | 1 | 1 | Cer(d18:1/14:0) |
| Triradylglycerols | 4 / 34200 | 4.66 | 0.708 | 1 | 1 | TG(16:0/16:0/16:0); TG(18:0/18:0/18:0); TG(20:0/20:0/20:0); TG(52:9) |
| Glycerophospho-  lipids | 4 / 36400 | 4.95 | 0.757 | 1 | 1 | PC(22:5(7Z,10Z,13Z,16Z,19Z)/18:4(6Z,9Z,12Z,15Z)); PI(16:0/18:1(9Z)); PI(16:0/20:4(5Z,8Z,11Z,14Z)); PI(18:1(9Z)/20:4(5Z,8Z,11Z,14Z)) |
| Glycosphingolipids | 1 / 13500 | 1.83 | 0.85 | 1 | 1 | GlcCer(d18:2/20:1) |
| Glycerolipids | 2 / 41400 | 5.63 | 0.985 | 1 | 1 | TG(16:0/16:0/18:0); TG(12:0/16:0/18:0) |


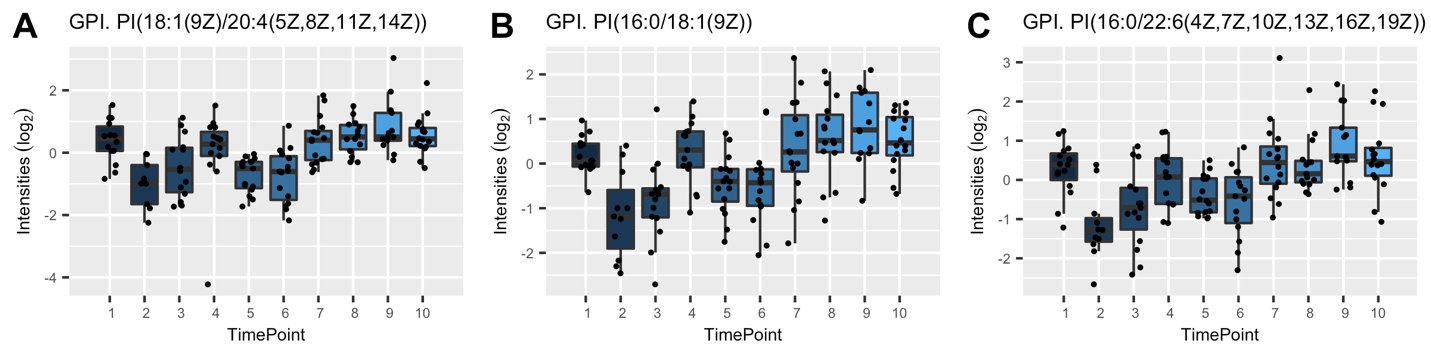


**Figure S3.** Boxplot of identified lipids within enriched lipid classes of Group 1 with a *P* value < .001. Only lipids with exact name matches have been included. GPI stands for glycerophosphoinositol. Intensities are standardized and on a log_2_ scale.

**Table ~~S7~~ S8.** Results from enrichment analysis of identified differentially expressed lipids within Group 2.

| **Lipid class** | **Hits / Total** | **Expected** | **Raw *P* value** | **Holm *P* value** | **FDR** | **Lipids** |
| --- | --- | --- | --- | --- | --- | --- |
| Ceramides | 10 / 5560 | 0.476 | 4.79e-12 | 1.17e-09 | 1.17e-09 | Cer(d18:1/12:0); Cer(d18:1/16:0); Cer(d18:1/20:0); Cer(d18:1/22:0); Cer(d18:1/24:1(15Z)); Cer(d18:1/14:0); Cer(d18:0/13:0); Cer(d19:1/14:0); Cer(d20:1/12:0); Cer(d22:1/14:0) |
| Glycerophospho-ethanolamines | 5 / 4350 | 0.373 | 2.51e-05 | 0.0061 | 0.00306 | PE(16:0/16:0); PE(16:0/20:5(5Z,8Z,11Z,14Z,17Z)); PE(18:0/22:6(4Z,7Z,10Z,13Z,16Z,19Z)); PE(22:2(13Z,16Z)/13:0); PE(P-18:0/17:0) |
| Glycerophospho-  cholines | 1 / 4700 | 0.403 | 0.335 | 1 | 1 | PC(35:3) |
| Glycerophospho-  lipids | 1 / 36400 | 3.12 | 0.968 | 1 | 1 | PC(P-16:0/P-16:0) |

**
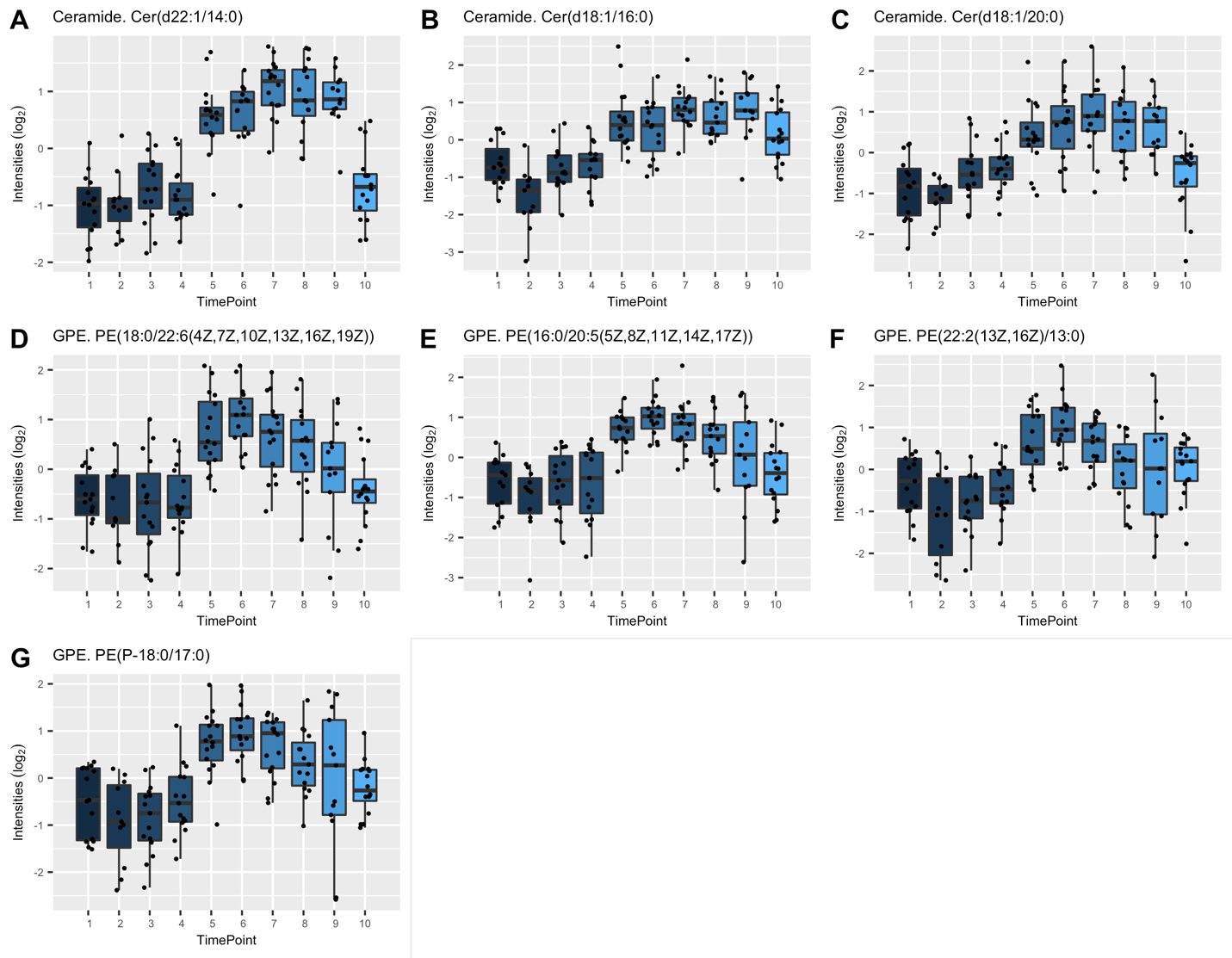
**

**Figure S4.** Boxplot of identified lipids within enriched lipid classes of Group 2 with a *P* value < .001. Only lipids with exact name matches have been included. GPE stands for glycerophosphoethanolamines. Intensities are standardized and on a log_2_ scale.

**Table ~~S8~~ S9.** Results from enrichment analysis of identified differentially expressed lipids within Group 3.

| **Lipid class** | **Hits / Total** | **Expected** | **Raw *P* value** | **Holm *P* value** | **FDR** | **Lipids** |
| --- | --- | --- | --- | --- | --- | --- |
| Glycerophospho-cholines | 9 / 4700 | 0.261 | 1.23e-13 | 3.01e-11 | 3.01e-11 | PC(16:0/18:1(9Z)); PC(22:0/18:3(6Z,9Z,12Z)); PC(12:0/13:0); PC(10:0/22:0); PC(10:0/23:0); PC(O-16:0/18:1(9Z)); PC(O-18:0/20:1(9Z)); PC(P-14:0/18:1(9Z)); PC(P-20:0/22:6(4Z,7Z,10Z,13Z,16Z,19Z)) |
| Phosphosphingo-  lipids | 2 / 4800 | 0.266 | 0.0278 | 1 | 1 | SM(d18:1/18:0); SM(d18:1/16:0) |
| Sphingomyelins | 1 / 2320 | 0.128 | 0.121 | 1 | 1 | SM(d18:1/16:0) |

###
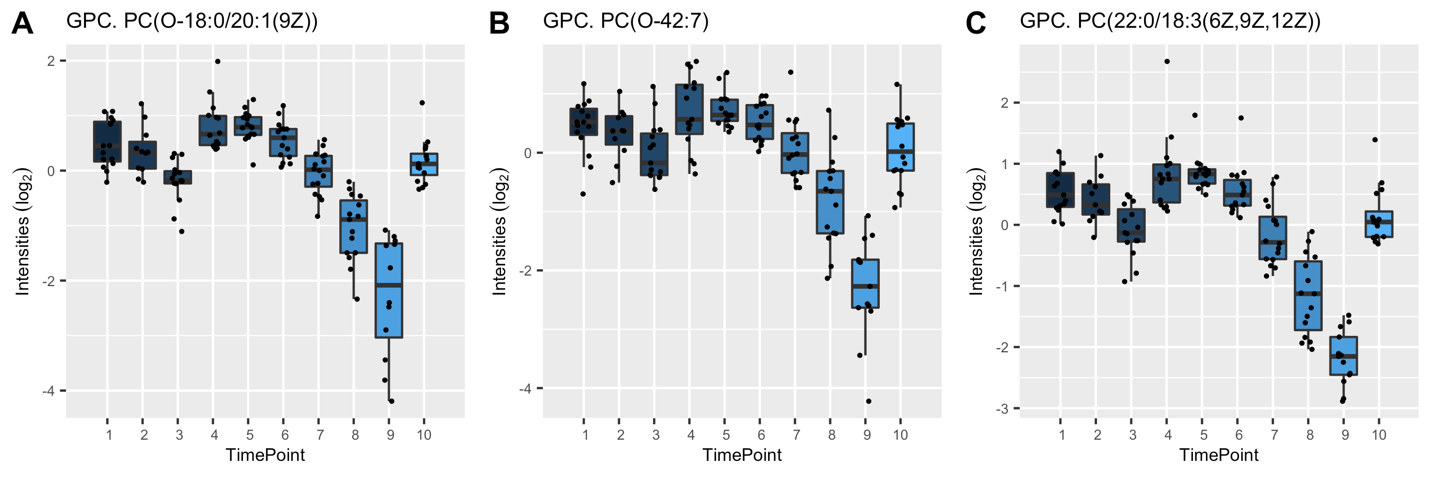


### Figure S5. Boxplot of identified lipids within enriched lipid classes of Group 3 with a *P* value < .001. Only lipids with exact name matches have been included. GPC stands for glycerophosphocholines. Intensities are standardized and on a log_2_ scale.

**Table ~~S9~~ S10.** Results from enrichment analysis of identified differentially expressed lipids within Group 5.

| **Lipid class** | **Hits / Total** | **Expected** | **Raw *P* value** | **Holm *P* value** | **FDR** | **Lipids** |
| --- | --- | --- | --- | --- | --- | --- |
| Glycerophospho-cholines | 7 / 4700 | 0.379 | 3.96e-08 | 9.66e-06 | 9.66e-06 | PC(16:0/16:0); PC(P-18:0/18:1(9Z)); PC(O-30:0); PC(o-18:0/20:4(8Z,11Z,14Z,17Z)); PC(o-18:0/22:6(4Z,7Z,10Z,13Z,16Z,19Z)); PC(O-16:0/18:1(9Z)); PC(P-18:0/22:1(11Z)) |
| Sphingoid bases | 2 / 98 | 0.0079 | 2.88e-05 | 0.00701 | 0.00352 | Sphinganine; Sphingosine |
| Sterols | 1 / 842 | 0.0679 | 0.0658 | 1 | 1 | episteryl oleate |
| Glycerophospho-  serines | 1 / 4140 | 0.334 | 0.286 | 1 | 1 | PS(34:2) |
| Glycerophospho-  glycerols | 1 / 4190 | 0.338 | 0.289 | 1 | 1 | PG(O-20:0/21:0) |
| Glycosphingo-  lipids | 2 / 13500 | 1.09 | 0.297 | 1 | 1 | LacCer(d18:1/24:0); GlcCer(d18:1/12:0) |
| Glycerophospho-  ethanolamines | 1 / 4350 | 0.351 | 0.299 | 1 | 1 | PE(P-18:0/24:0) |
| Glycerolipids | 1 / 41400 | 3.34 | 0.976 | 1 | 1 | TG(12:0/16:0/18:0) |


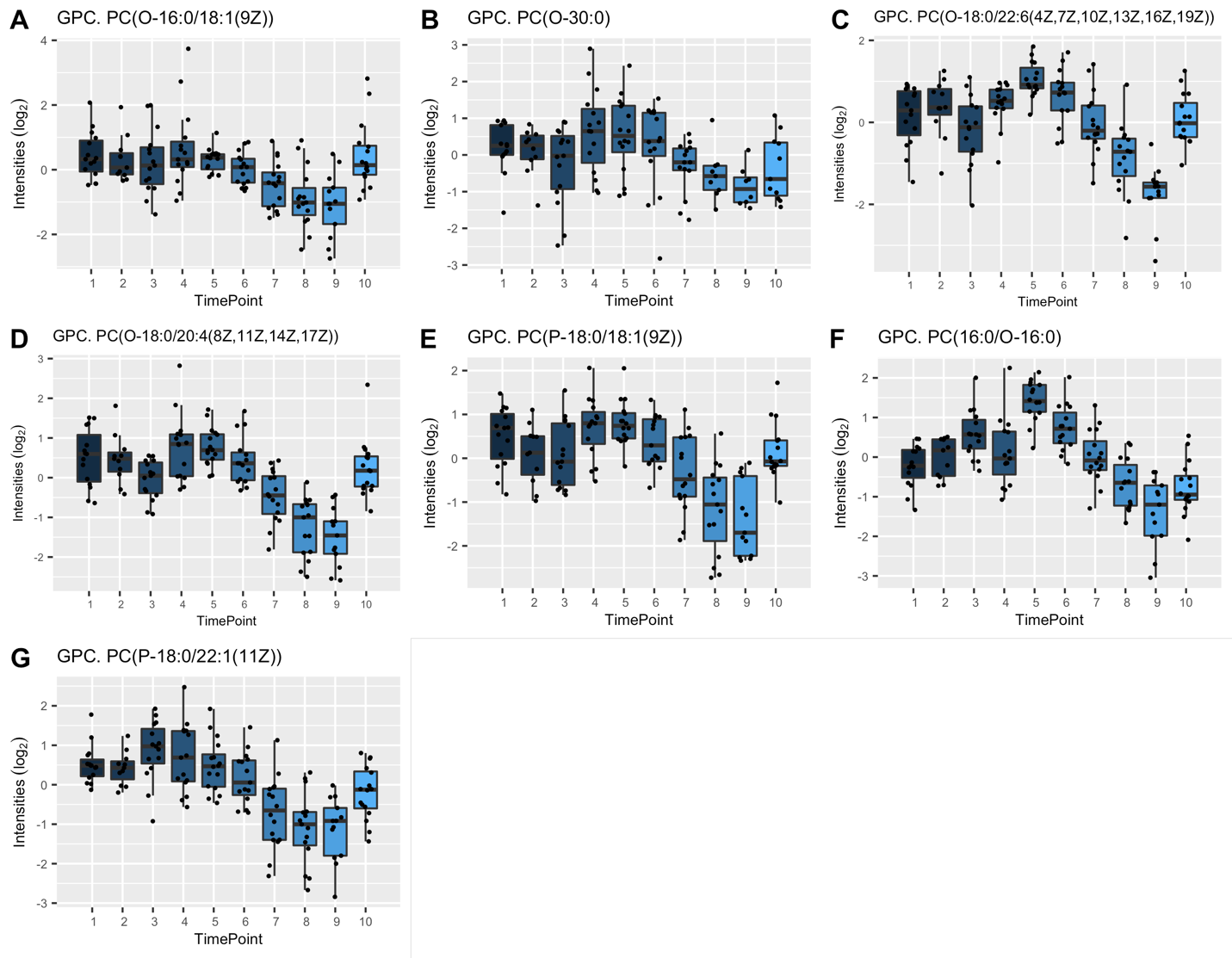


**Figure S6.** Boxplot of identified lipids within enriched lipid classes of Group 5 with a *P* value < .001. Only lipids with exact name matches have been included. GPC stands for glycerophosphocholines. Intensities are standardized and on a log_2_ scale.

**Table ~~S10~~ S11.** Results from enrichment analysis of identified differentially expressed lipids within Group 6.

| **Lipid class** | **Hits / Total** | **Expected** | **Raw *P* value** | **Holm *P* value** | **FDR** | **Lipids** |
| --- | --- | --- | --- | --- | --- | --- |
| Glycosphingo-  lipids | 3 / 13500 | 0.476 | 0.00891 | 1 | 1 | Lactosylceramide (d18:1/24:1(15Z)); Glucosylceramide (d18:1/20:0); Glucosylceramide (d18:1/22:0) |
| Glycerophospho-cholines | 2 / 4700 | 0.166 | 0.0109 | 1 | 1 | PC(16:0/18:1(9Z)); PC(P-18:0/22:0) |
| Fatty Acids and Conjugates | 1 / 3090 | 0.109 | 0.104 | 1 | 1 | Arachidonic acid |
| Ceramides | 1 / 5560 | 0.196 | 0.18 | 1 | 1 | CerP(d16:1/14:0) |
| Glycerophospho-  lipids | 1 / 36400 | 1.28 | 0.758 | 1 | 1 | PC(P-18:0/22:0) |


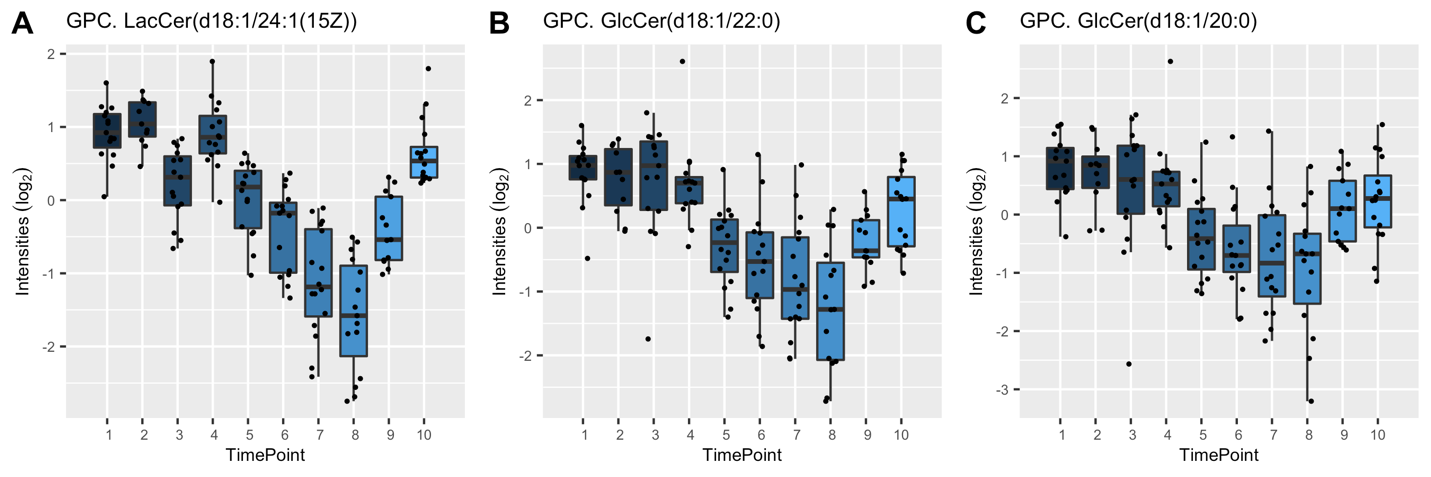


**Figure S7.** Boxplot of identified lipids within enriched lipid classes of G6 with a *P* value < .001. Only lipids with exact name matches have been included. GPC stands for glycerophosphocholines. Intensities are standardized and on a log_2_ scale.


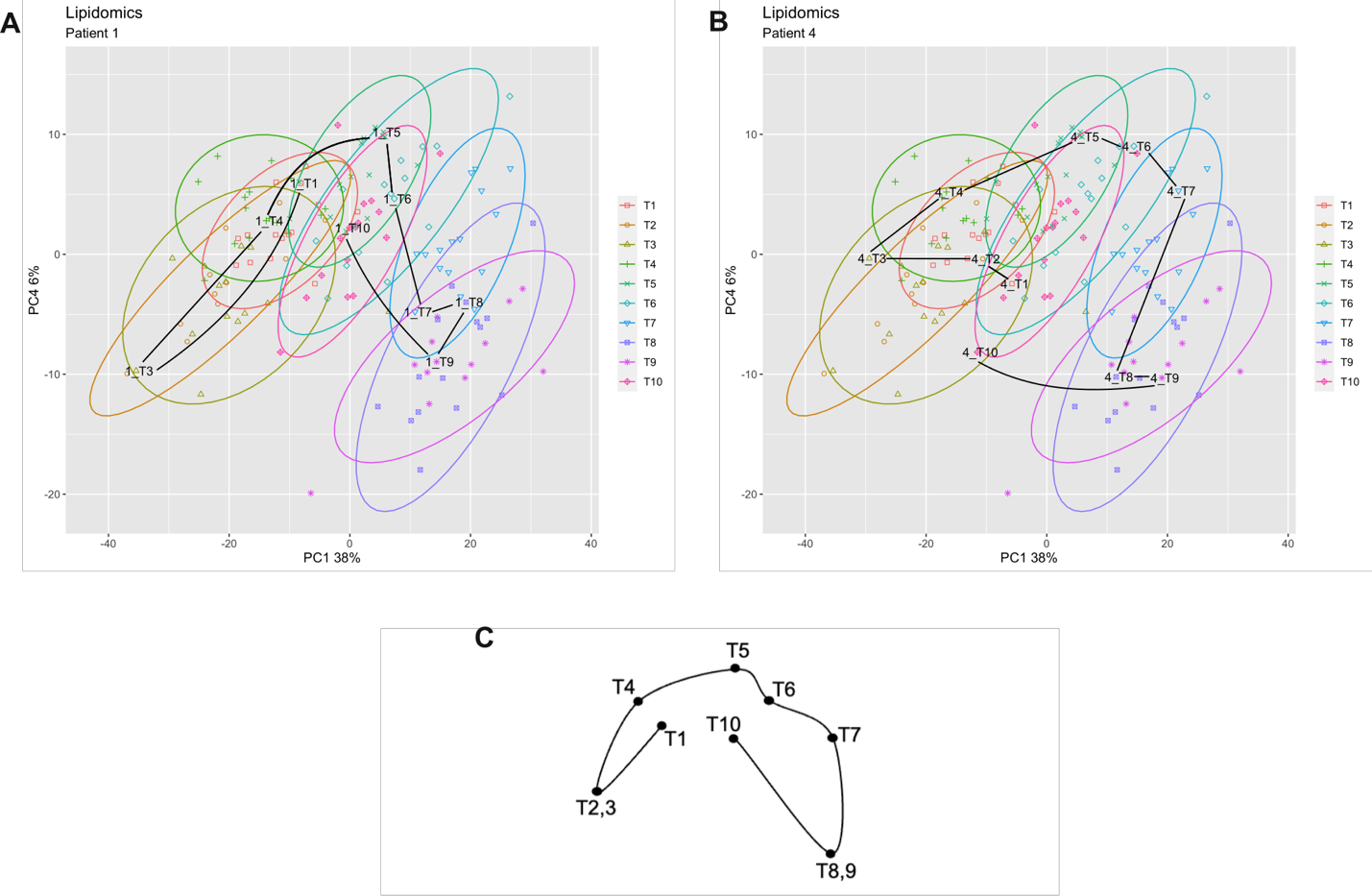


**Figure S8.** PCA depicting PC1 and PC4 obtained from differentially expressed features within the lipidomics data from RRMS patients undergoing AHSCT. The percentage explained variance is presented on the corresponding axis. (A) Illustrates the data points for patient 1 where EDA occurred after 1 year. (B) Illustrates the data points for patient 4 where EDA failure occurred after 2 years. (C) Depicts the mean PC1 for all 16 patients undergoing AHSCT.


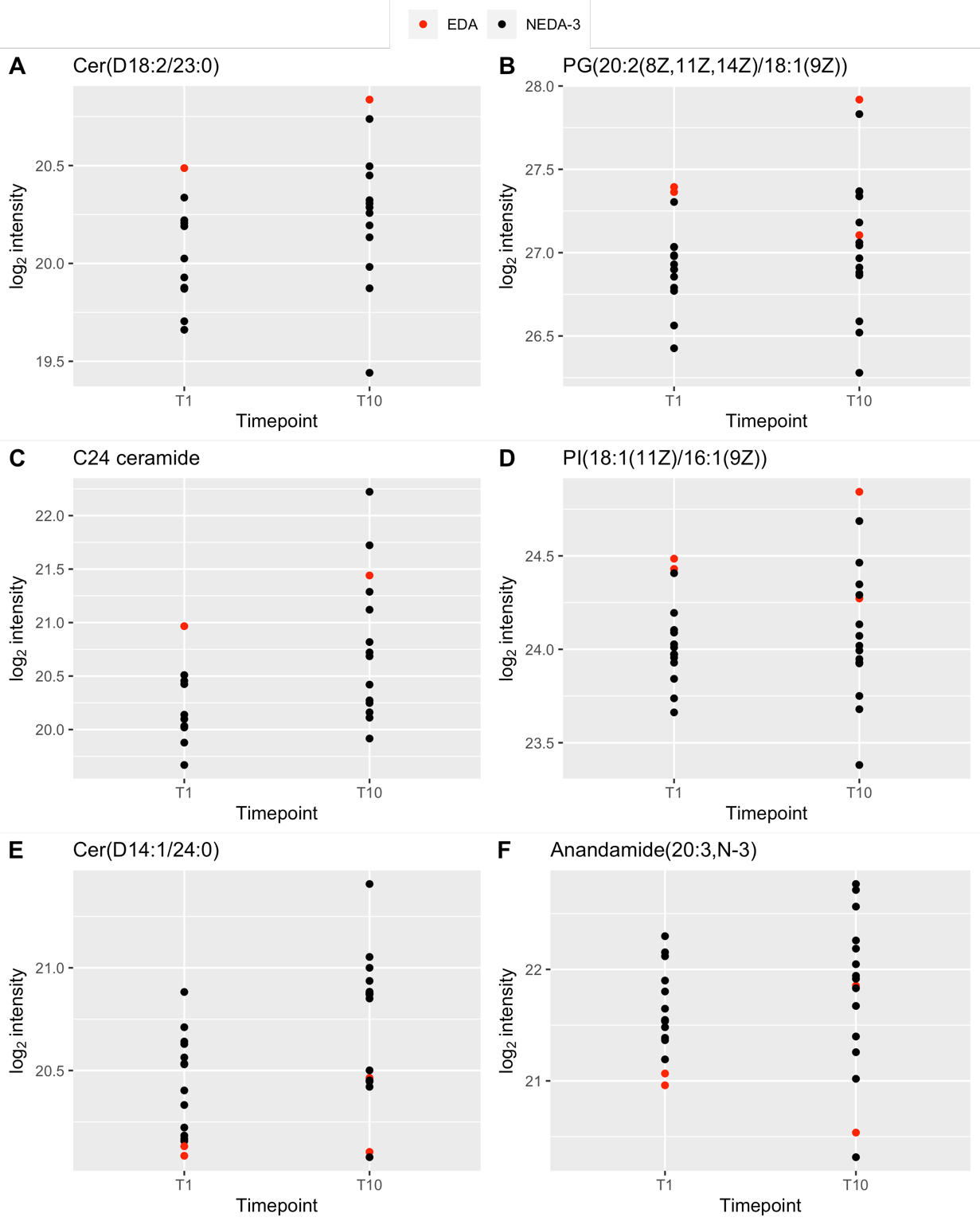


**Figure S9.** Lipid levels prior to and after AHSCT for lipids deviating between patients with EDA within three years after treatment and patients with NEDA-3 after three years. (A-D) Identified lipids where patients with EDA had higher lipid levels at T1 compared to patients with NEDA-3 three years after treatment. (E-F) Identified lipids where patients with EDA had lower lipid levels at T1 compared to patients with NEDA-3 three years after treatment


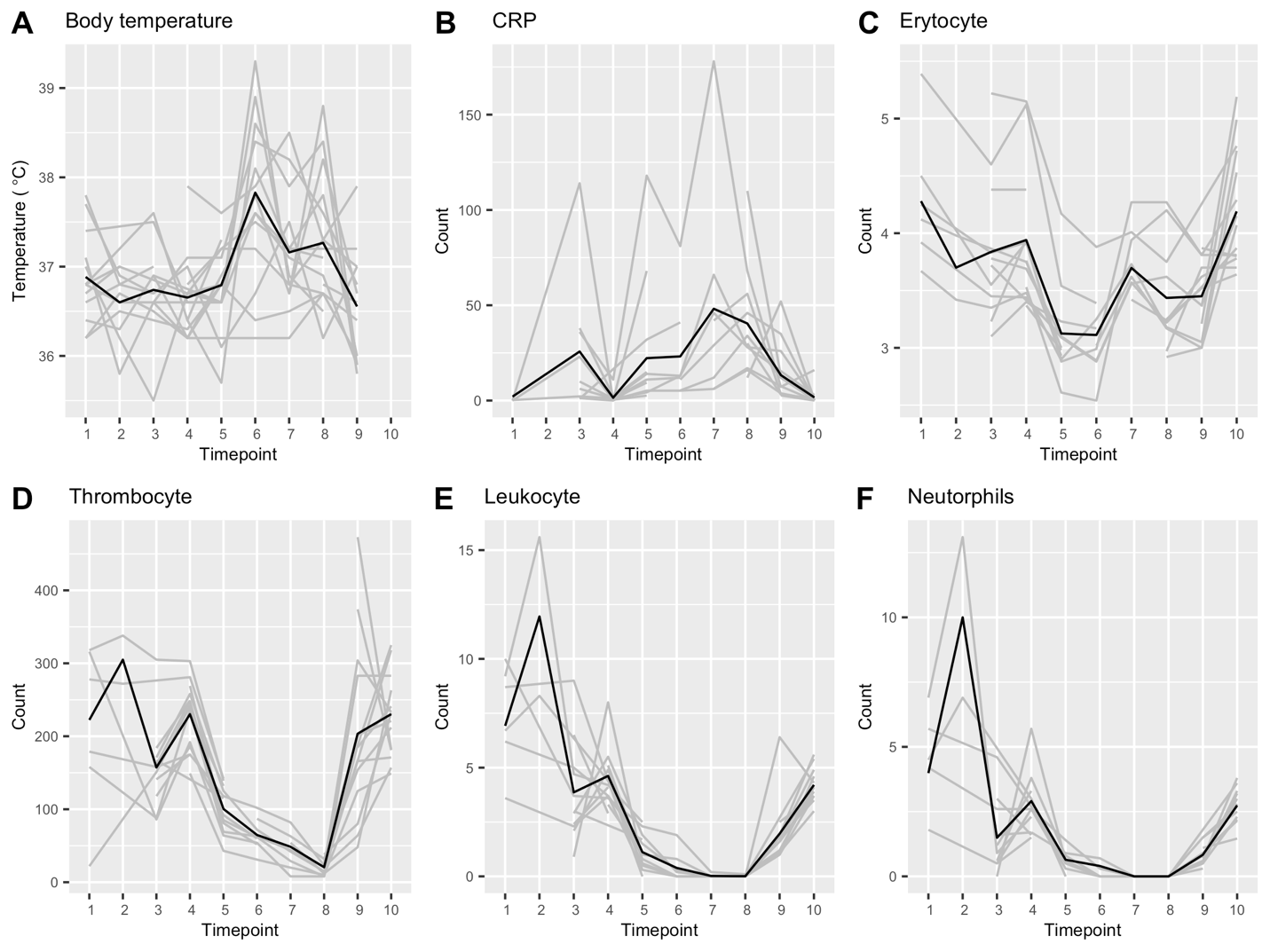


**Figure ~~S8~~ S10.** Expression profiles of the six clinical measurements analyzed during AHSCT. The six clinical measurements were: body temperature, C-reactive protein (CRP), erytocyte, thrombocyte, leukocyte, and neutrophil counts. The gray curves are the individual curves for each RRMS patient, while the black is the mean curve.

*
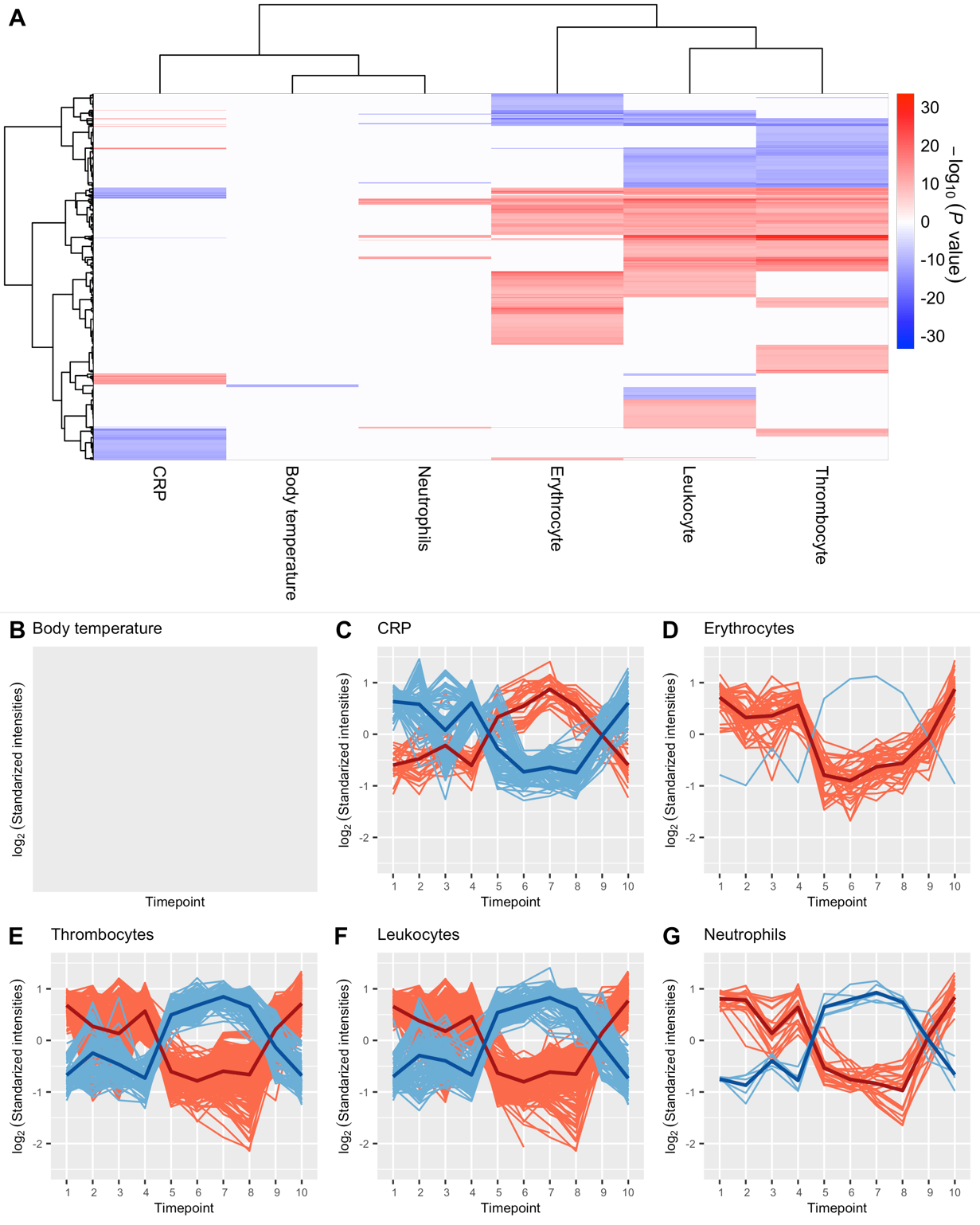
*

**Figure ~~S9~~ S11.** Metabolite association to clinical measurements through AHSCT. (A) Heatmap over associated metabolites to the six clinical measurements: C-reactive protein (CRP), body temperature, neutrophil, erythrocyte, leukocyte, and thrombocyte count. No metabolites were associated with body temperature. (B to F) The expression profiles of the associated metabolites. Blue represents negatively associated metabolite and red positively associated metabolite. A mean curve has been drawn for each association type.

**Table ~~S11~~ S12.** Results from enrichment analysis of identified lipids associated with C-reactive protein (CRP).

| **Lipid class** | **Hits / Total** | **Expected** | **Raw *P* value** | **Holm *P* value** | **FDR** | **Lipids** |
| --- | --- | --- | --- | --- | --- | --- |
| Glycerophospho-cholines | 7 / 4700 | 0.498 | 3.63e-07 | 8.85e-05 | 8.85e-05 | PC(16:0/18:1(9Z)); PC(20:4(5Z,8Z,11Z,14Z)/20:4(5Z,8Z,11Z,14Z)); LysoPC(20:0/0:0); LysoPC(22:0); PC(24:0); PC(O-16:0/18:1(9Z)); PC(P-14:0/18:1(9Z)) |
| Glycerophospho-ethanolamines | 6 / 4350 | 0.46 | 4.52e-06 | 0.0011 | 0.000552 | PE(16:1(9Z)/22:2(13Z,16Z)); PE(P-16:0/18:2(9Z,12Z)); PE(P-18:0/18:2(9Z,12Z)); PE(P-18:1(9Z)/18:1(9Z)); PE(P-18:1(9Z)/18:2(9Z,12Z)); PE(P-20:0/17:2(9Z,12Z)) |
| Sphingolipids | 1 / 487 | 0.0515 | 0.0503 | 1 | 1 | Glucosylsphingosine |
| Acidic glycosphingolipids | 1 / 511 | 0.0541 | 0.0527 | 1 | 1 | (3'-sulfo)Galbeta-Cer(d18:1/24:1(15Z)(2OH)) |
| Sterols | 1 / 842 | 0.0891 | 0.0854 | 1 | 1 | Desmosterol |
| Phosphosphingo-lipids | 1 / 4800 | 0.508 | 0.402 | 1 | 1 | SM(d18:1/18:0) |
| Glycosphingolipids | 2 / 13500 | 1.43 | 0.423 | 1 | 1 | Glucosylceramide (d18:1/20:0); Glucosylceramide (d18:1/22:0) |
| Ceramides | 1 / 5560 | 0.588 | 0.449 | 1 | 1 | Cer(d18:0/22:0) |
| Glycerophospho-lipids | 1 / 36400 | 3.85 | 0.986 | 1 | 1 | PC(22:5(7Z,10Z,13Z,16Z,19Z)/20:5(5Z,8Z,11Z,14Z,17Z)) |

**Table ~~S12~~ S13.** Results from enrichment analysis of identified lipids associated with erythrocyte count.

| **Lipid class** | **Hits / Total** | **Expected** | **Raw *P* value** | **Holm *P* value** | **FDR** | **Lipids** |
| --- | --- | --- | --- | --- | --- | --- |
| Glycerophospho-ethanolamines | 14 / 4350 | 1.21 | 1.09e-11 | 2.65e-09 | 2.65e-09 | PE(18:1(9Z)/22:2(13Z,16Z)); PE(P-16:0/20:4(5Z,8Z,11Z,14Z)); PE(P-18:0/18:1(9Z)); PE(P-18:0/18:3(9Z,12Z,15Z)); PE(P-18:0/22:4(7Z,10Z,13Z,16Z)); PE(P-18:1(9Z)/18:1(9Z)); PE(P-18:1(9Z)/18:2(9Z,12Z)); PE(20:0/21:0); PE(P-16:0/19:1(9Z)); PE(P-18:0/17:2(9Z,12Z)); PE(P-20:0/17:2(9Z,12Z)); PE(P-20:0/18:1(9Z)); PE(P-20:0/18:3(9Z,12Z,15Z)); PE(P-20:0/20:0) |
| Ceramides | 10 / 5560 | 1.54 | 2.72e-06 | 6.62e-04 | 3.32e-04 | Ceramide (d18:1/20:0); Cer(d18:0/22:0); Cer(d18:0/23:0); Cer(d18:1/14:0); Cer(d19:0/23:0); Cer(d20:0/22:0); Cer(d20:1/24:0); Cer(d20:1/25:0); Cer(d18:0/h24:0); Cer(d18:0/h26:0) |
| Glycerophospho-cholines | 9 / 4700 | 1.3 | 5.56e-06 | 0.00134 | 4.52e-04 | PC(15:0/22:2(13Z,16Z)); PC(16:0/18:1(9Z)); PC(16:0/22:4(7Z,10Z,13Z,16Z)); PC(18:0/18:0); PC(20:4(5Z,8Z,11Z,14Z)/20:4(5Z,8Z,11Z,14Z)); PC(12:0/13:0); PC(16:1(9Z)/19:0); PC(18:4(6Z,9Z,12Z,15Z)/21:0); PC(22:6(4Z,7Z,10Z,13Z,16Z,19Z)/17:0) |
| Sphingolipids | 3 / 487 | 0.135 | 3.5e-04 | 0.0844 | 0.0214 | SM(d18:1/24:0); SM(d18:0/24:1(15Z)(OH)); AS 1-5 |
| Quinones and hydroquinones | 1 / 63 | 0.0175 | 0.0173 | 1 | 0.845 | Vitamin K2 |
| Sphingoid bases | 1 / 98 | 0.0272 | 0.0268 | 1 | 1 | Phytosphingosine |
| Steroid conjugates | 1 / 123 | 0.0341 | 0.0335 | 1 | 1 | 5a-Dihydrotestosterone sulfate |
| Fatty Acids and Conjugates | 3 / 3090 | 0.856 | 0.0543 | 1 | 1 | Undecylenic acid; 3-Oxohexacosanoic acid; 27:3(5Z,9Z,20Z) |
| Fatty amides | 1 / 406 | 0.113 | 0.107 | 1 | 1 | N-arachidonoyl glycine |
| Acidic glycosphingolipids | 1 / 511 | 0.142 | 0.132 | 1 | 1 | (3'-sulfo)Galbeta-Cer(d18:1/24:1(15Z)(2OH)) |
| Sphingomyelins | 2 / 2320 | 0.642 | 0.135 | 1 | 1 | SM(d18:1/21:0); SM(d18:1/24:0) |
| Sterols | 1 / 842 | 0.233 | 0.209 | 1 | 1 | Epidiosgenin |
| Isoprenoids | 1 / 1420 | 0.393 | 0.326 | 1 | 1 | Pfaffic acid |
| Glycerophospho-serines | 1 / 4140 | 1.15 | 0.687 | 1 | 1 | PS(46:4) |
| Glycerophospho-inositols | 1 / 4360 | 1.21 | 0.705 | 1 | 1 | PI(P-20:0/17:0) |
| Phosphosphingo-lipids | 1 / 4800 | 1.33 | 0.74 | 1 | 1 | MIPC(d20:0/24:0) |
| Glycerophospho-lipids | 4 / 36400 | 10.1 | 0.994 | 1 | 1 | PC(22:5(7Z,10Z,13Z,16Z,19Z)/18:2(9Z,12Z)); PE(P-18:0/22:5(7Z,10Z,13Z,16Z,19Z)); PE(P-18:1(11Z)/20:1(11Z)); PE(P-18:1(9Z)/20:4(5Z,8Z,11Z,14Z)) |
| Glycerolipids | 1 / 41400 | 11.5 | 1 | 1 | 1 | TG(12:0/16:0/18:0) |

**Table ~~S13~~ S14.** Results from enrichment analysis of identified lipids associated with thrombocyte count.

| **Lipid class** | **Hits / Total** | **Expected** | **Raw *P* value** | **Holm *P* value** | **FDR** | **Lipids** |
| --- | --- | --- | --- | --- | --- | --- |
| Glycerophospho-cholines | 12 / 4700 | 1.35 | 8.13e-09 | 1.98e-06 | 1.98e-06 | PC(14:0/18:1(11Z)); PC(15:0/22:2(13Z,16Z)); PC(16:0/18:1(9Z)); PC(18:0/18:0); PC(18:0/22:6(4Z,7Z,10Z,13Z,16Z,19Z)); PC(20:4(5Z,8Z,11Z,14Z)/20:4(5Z,8Z,11Z,14Z)); LysoPC(18:0); LysoPC(20:0/0:0); LysoPC(20:1(11Z)); LysoPC(22:0); PC(O-30:0); PC(16:1(9Z)/19:0) |
| Ceramides | 9 / 5560 | 1.6 | 2.81e-05 | 0.00684 | 0.00343 | Cer(d18:1/12:0); Cer(d18:0/22:0); Cer(d18:0/23:0); Cer(d18:1/14:0); Cer(d15:0/25:0); Cer(d19:0/23:0); Cer(d20:0/22:0); CerP(d16:1/14:0); Cer(t18:0/24:0) |
| Glycerophospho-ethanolamines | 5 / 4350 | 1.25 | 0.00823 | 1 | 0.67 | PE(14:0/20:1(11Z)); PE(16:0/20:4(5Z,8Z,11Z,14Z)); PE(16:0/20:5(5Z,8Z,11Z,14Z,17Z)); PE(P-18:0/22:4(7Z,10Z,13Z,16Z)); PE(P-20:0/18:1(9Z)) |
| Quinones and hydroquinones | 1 / 63 | 0.0181 | 0.0179 | 1 | 1 | alpha-Tocopherol |
| Sterol esters | 1 / 86 | 0.0247 | 0.0244 | 1 | 1 | CE(12:0) |
| Sphingoid bases | 1 / 98 | 0.0281 | 0.0278 | 1 | 1 | Sphinganine |
| Glycerophospho-serines | 4 / 4140 | 1.19 | 0.0312 | 1 | 1 | PS(37:0); PS(46:4); PS(21:0/15:0); PS(21:0/20:1(11Z)) |
| Steroid conjugates | 1 / 123 | 0.0353 | 0.0347 | 1 | 1 | 5a-Dihydrotestosterone sulfate |
| Oxidized glycerophospho-lipids | 1 / 244 | 0.0701 | 0.0677 | 1 | 1 | PKODiA-PE |
| Bile acids | 1 / 311 | 0.0893 | 0.0855 | 1 | 1 | N-[(3a,5b,7a)-3-hydroxy-24-oxo-7-(sulfooxy)cholan-24-yl]-Glycine |
| Fatty esters | 2 / 1870 | 0.538 | 0.101 | 1 | 1 | L-Hexanoylcarnitine; Stearoylcarnitine |
| Sphingolipids | 1 / 487 | 0.14 | 0.131 | 1 | 1 | AS 1-5 |
| Acidic glycosphingolipids | 1 / 511 | 0.147 | 0.137 | 1 | 1 | (3'-sulfo)Galbeta-Cer(d18:1/24:1(15Z)(2OH)) |
| Sphingomyelins | 2 / 2320 | 0.665 | 0.143 | 1 | 1 | SM(d18:0/22:0); SM(d18:1/21:0) |
| Steroids | 1 / 1050 | 0.301 | 0.26 | 1 | 1 | Epiandrosterone |
| Glycosphingolipids | 4 / 13500 | 3.87 | 0.547 | 1 | 1 | LacCer(d18:1/24:1(15Z)); Glucosylceramide (d18:1/20:0); Glucosylceramide (d18:1/22:0); LacCer(d18:1/14:0) |
| Glycerophospho-inositols | 1 / 4360 | 1.25 | 0.718 | 1 | 1 | PI(P-20:0/17:0) |
| Phosphosphingo-lipids | 1 / 4800 | 1.38 | 0.752 | 1 | 1 | SM(d18:1/18:0) |
| Glycerophospho-lipids | 7 / 36400 | 10.5 | 0.919 | 1 | 1 | PC(18:3(6Z,9Z,12Z)/24:0); PC(22:5(7Z,10Z,13Z,16Z,19Z)/18:0); PC(22:5(7Z,10Z,13Z,16Z,19Z)/20:0); PC(22:5(7Z,10Z,13Z,16Z,19Z)/20:5(5Z,8Z,11Z,14Z,17Z)); PGP(18:1(9Z)/22:4(7Z,10Z,13Z,16Z)); PS(MonoMe(11,5)/DiMe(11,3)); 1-Stearoylglycerophosphoserine |
| Glycerolipids | 1 / 41400 | 11.9 | 1 | 1 | 1 | TG(16:0/16:0/18:0) |

**Table ~~S14~~ S15.** Results from enrichment analysis of identified lipids associated with leukocyte count.

| **Lipids class** | **Hits / Total** | **Expected** | **Raw *P* value** | **Holm *P* value** | **FDR** | **Lipids** |
| --- | --- | --- | --- | --- | --- | --- |
| Ceramides | 14 / 5560 | 1.68 | 9.3e-10 | 2.27e-07 | 2.27e-07 | Cer(d18:1/12:0); Cer(d18:1/20:0); Cer(d18:0/16:0); Cer(d18:0/22:0); Cer(d18:0/25:0); Cer(d18:0/26:0); Cer(d18:1/14:0); Cer(d15:0/25:0); Cer(d16:1/22:0); Cer(d19:0/23:0); Cer(d20:0/22:0); Cer(d20:1/25:0); Cer(d22:1/14:0); CerP(d16:1/14:0) |
| Glycerophospho-cholines | 10 / 4700 | 1.42 | 1.41e-06 | 0.000344 | 0.000173 | PC(15:0/22:2(13Z,16Z)); PC(16:0/18:1(9Z)); PC(18:0/18:0); PC(18:0/22:6(4Z,7Z,10Z,13Z,16Z,19Z)); PC(20:4(5Z,8Z,11Z,14Z)/20:4(5Z,8Z,11Z,14Z)); LysoPC(20:0/0:0); PC(O-30:0); PC(16:1(9Z)/19:0); PC(19:0/22:6(4Z,7Z,10Z,13Z,16Z,19Z)); PC(19:0/0:0) |
| Sphingolipids | 3 / 487 | 0.147 | 0.000453 | 0.11 | 0.0368 | Galabiosylceramide (d18:1/22:0); SM(d18:0/24:1(15Z)(OH)); AS 1-5 |
| Fatty amides | 2 / 406 | 0.123 | 0.00683 | 1 | 0.417 | N-arachidonoyl glycine; N-ethyl arachidonoyl amine |
| Quinones and hydroquinones | 1 / 63 | 0.019 | 0.0189 | 1 | 0.921 | Vitamin K2 |
| Sterol esters | 1 / 86 | 0.026 | 0.0257 | 1 | 1 | CE(12:0) |
| Sphingoid bases | 1 / 98 | 0.0296 | 0.0292 | 1 | 1 | Sphinganine |
| Steroid conjugates | 1 / 123 | 0.0372 | 0.0365 | 1 | 1 | 5a-Dihydrotestosterone sulfate; |
| Glycerophospho-ethanolamines | 4 / 4350 | 1.32 | 0.0427 | 1 | 1 | PE(P-18:0/18:3(9Z,12Z,15Z)); PE(P-18:0/22:4(7Z,10Z,13Z,16Z)); PE(P-18:1(9Z)/18:2(9Z,12Z)); PE(P-20:0/18:1(9Z)) |
| Bile acids | 1 / 311 | 0.094 | 0.0898 | 1 | 1 | N-[(3a,5b,7a)-3-hydroxy-24-oxo-7-(sulfooxy)cholan-24-yl]-Glycine |
| Acidic glycosphingolipids | 1 / 511 | 0.154 | 0.143 | 1 | 1 | (3'-sulfo)Galbeta-Cer(d18:1/24:1(15Z)(2OH)) |
| Neutral glycosphingolipids | 1 / 532 | 0.161 | 0.149 | 1 | 1 | GlcCer(d18:1/16:0) |
| Sphingomyelins | 2 / 2320 | 0.7 | 0.155 | 1 | 1 | SM(d18:1/12:0); SM(d18:1/21:0) |
| Steroids | 1 / 1050 | 0.317 | 0.272 | 1 | 1 | Testosterone |
| Isoprenoids | 1 / 1420 | 0.429 | 0.35 | 1 | 1 | Pfaffic acid |
| Phosphosphingo-lipids | 2 / 4800 | 1.45 | 0.427 | 1 | 1 | SM(d18:1/18:0); MIPC(d20:0/24:0) |
| Fatty esters | 1 / 1870 | 0.566 | 0.434 | 1 | 1 | Stearoylcarnitine |
| Prenol lipids | 1 / 2470 | 0.746 | 0.528 | 1 | 1 | Vinaginsenoside R13 |
| Fatty Acids and Conjugates | 1 / 3090 | 0.933 | 0.61 | 1 | 1 | 3-Oxohexacosanoic acid |
| Glycerophospho-serines | 1 / 4140 | 1.25 | 0.718 | 1 | 1 | PS(21:0/15:0) |
| Glycerophospho-inositols | 1 / 4360 | 1.32 | 0.736 | 1 | 1 | PI(P-20:0/17:0) |
| Glycosphingolipids | 2 / 13500 | 4.08 | 0.921 | 1 | 1 | Lactosylceramide (d18:1/24:1(15Z)); LacCer(d18:1/14:0) |
| Glycerophospho-lipids | 5 / 36400 | 11 | 0.991 | 1 | 1 | PC(22:5(7Z,10Z,13Z,16Z,19Z)/18:2(9Z,12Z)); PC(22:5(7Z,10Z,13Z,16Z,19Z)/20:5(5Z,8Z,11Z,14Z,17Z)); PE(P-18:1(11Z)/20:1(11Z)); PE(P-18:1(9Z)/20:4(5Z,8Z,11Z,14Z)); PS(MonoMe(11,5)/DiMe(11,3)) |
| Glycerolipids | 2 / 41400 | 12.5 | 1 | 1 | 1 | DG(21:0/8:0/0:0); TG(12:0/16:0/18:0) |

**Table ~~S15~~ S16.** Results from enrichment analysis of identified lipids associated with neutrophil count.

| **Lipid class** | **Hits / Total** | **Expected** | **Raw *P* value** | **Holm *P* value** | **FDR** | **Lipids** |
| --- | --- | --- | --- | --- | --- | --- |
| Sphingoid bases | 1 / 98 | 0.00247 | 0.00247 | 0.602 | 0.602 | Sphinganine |
| Sphingolipids | 1 / 487 | 0.0123 | 0.0122 | 1 | 1 | SM(d18:0/24:1(15Z)(OH)) |
| Phosphosphingo-lipids | 1 / 4800 | 0.121 | 0.115 | 1 | 1 | SM(d18:1/18:0) |
| Ceramides | 1 / 5560 | 0.14 | 0.132 | 1 | 1 | Cer(d18:1/14:0) |
| Glycosphingolipids | 1 / 13500 | 0.34 | 0.297 | 1 | 1 | Lactosylceramide (d18:1/24:1(15Z)) |
